# Associations between daily air quality and hospitalisations for acute exacerbation of chronic obstructive pulmonary disease in Beijing, 2013–17: an ecological analysis

**DOI:** 10.1016/S2542-5196(19)30085-3

**Published:** 2019-06

**Authors:** Lirong Liang, Yutong Cai, Benjamin Barratt, Baolei Lyu, Queenie Chan, Anna L Hansell, Wuxiang Xie, Di Zhang, Frank J Kelly, Zhaohui Tong

**Affiliations:** a Clinical Epidemiology and Tobacco Dependence Treatment Research Department, Beijing Institute of Respiratory Medicine, Beijing Chaoyang Hospital, Capital Medical University, Beijing, China; b Department of Respiratory and Critical Care Medicine, Beijing Institute of Respiratory Medicine, Beijing Chaoyang Hospital, Capital Medical University, Beijing, China; c MRC-PHE Centre for Environment and Health, Department of Analytical, Environmental and Forensic Sciences, School of Population Health and Environmental Sciences, King's College London, London, UK; d Department of Epidemiology and Biostatistics, School of Public Health, Imperial College London, London, UK; e Huayun Sounding (Beijing) Meteorological Technology Co, Beijing, China; f Centre for Environmental Health and Sustainability, University of Leicester, Leicester, UK; g Peking University Clinical Research Institute, Peking University Health Science Centre, Beijing, China

## Abstract

**Background:**

Air pollution in Beijing has been improving through implementation of the Air Pollution Prevention and Control Action Plan (2013–17), but its implications for respiratory morbidity have not been directly investigated. We aimed to assess the potential effects of air-quality improvements on respiratory health by investigating the number of cases of acute exacerbations of chronic obstructive pulmonary disease (COPD) advanced by air pollution each year.

**Methods:**

Daily city-wide concentrations of PM_10_, PM_2·5_, PM_coarse_ (particulate matter >2·5–10 μm diameter), nitrogen dioxide (NO_2_), sulphur dioxide (SO_2_), carbon monoxide (CO), and ozone (O_3_) in 2013–17 were averaged from 35 monitoring stations across Beijing. A generalised additive Poisson time-series model was applied to estimate the relative risks (RRs) and 95% CIs for hospitalisation for acute exacerbation of COPD associated with pollutant concentrations.

**Findings:**

From Jan 18, 2013, to Dec 31, 2017, 161 613 hospitalisations for acute exacerbation of COPD were recorded. Mean ambient concentrations of SO_2_ decreased by 68% and PM_2·5_ decreased by 33% over this 5-year period. For each IQR increase in pollutant concentration, RRs for same-day hospitalisation for acute exacerbation of COPD were 1·029 (95% CI 1·023–1·035) for PM_10_, 1·028 (1·021–1·034) for PM_2·5_, 1·018 (1·013–1·022) for PM_coarse_, 1·036 (1·028–1·044) for NO_2_, 1·019 (1·013–1·024) for SO_2_, 1·024 (1·018–1·029) for CO, and 1·027 (1·010–1·044) for O_3_ in the warm season (May to October). Women and patients aged 65 years or older were more susceptible to the effects of these pollutants on hospitalisation risk than were men and patients younger than 65 years. In 2013, there were 12 679 acute exacerbations of COPD cases that were advanced by PM_2·5_ pollution above the expected number of cases if daily PM_2·5_ concentrations had not exceeded the WHO target (25 μg/m^3^), whereas the respective figure in 2017 was 7377 cases.

**Interpretation:**

Despite improvement in overall air quality, increased acute air pollution episodes were significantly associated with increased hospitalisations for acute exacerbations of COPD in Beijing. Stringent air pollution control policies are important and effective for reducing COPD morbidity, and long-term multidimensional policies to safeguard public health are indicated.

**Funding:**

UK Medical Research Council.

## Introduction

Chronic obstructive pulmonary disease (COPD) is a leading contributor to disease burden globally.^1^ A survey in China^2^ showed an estimated nationwide prevalence of spirometry-defined COPD of 13·7% among people aged 40 years and older in 2012–15, a 5·5% increase from the 2002–04 survey, and suggested ambient air pollution resulting from rapid urbanisation as a probable contributor to this emerging COPD epidemic.

Although ambient air pollution might contribute to a more rapid decline of lung function and subsequent onset of COPD in adults,^3^, ^4^ its adverse effects on patients with existing COPD have also been widely reported. Short-term exposure to air pollution has been positively associated with COPD-related emergency department visits, hospital admissions, and mortality.^5^, ^6^ However, not all of these studies have specifically focused on acute exacerbations of COPD, an outcome that is often associated with COPD progression and prognosis, repeated access to health care, impaired quality of life, and mortality. A systematic review^6^ of 46 studies on acute exacerbations of COPD published until 2015 revealed heterogeneous evidence across studies in geographically diverse regions, but included few epidemiological studies from regions with severe ambient air pollution. Increasing numbers of studies have been reported from such regions in the past 4 years regarding short-term and long-term associations between air pollution and respiratory morbidity and mortality to help strengthen the evidence base.^1^, ^5^, ^7^, ^8^ Additionally, from both clinical and policy perspectives, important knowledge gaps remain concerning the exposure–response relation-ships between extremely high concentrations of ambient air pollution and risk of hospitalisation for acute exacerbation of COPD.

**Research in context**

**Evidence before this study**

We searched PubMed to identify systematic reviews of epidemiological studies on short-term air pollution and hospital admissions for chronic obstructive pulmonary disease (COPD), using a combination of search terms: “air pollution”, “COPD”, “hospital admission”, and “meta-analysis” or “systematic review”. The search was limited to studies that pooled results on a global scale, reported analytical pooled estimates, were written in English, and were published between Jan 1, 2013, and Dec 31, 2017. According to systematic reviews, numerous studies have reported that short-term increases in air pollution are significantly associated with COPD morbidity. However, the evidence was heterogeneous across studies in geographically diverse regions. Air pollution health responses in the Chinese population might differ from those of European and North American studies because of differences in the air pollution mixture and underlying health statuses of these populations. In 2013, the Air Pollution Prevention and Control Action Plan (APPCAP) was launched by the Chinese Government to curb air pollution to interim target concentrations by 2017. Reports have indicated that air quality has been improving during this period in China. However, no Chinese studies to date have directly investigated the implications of APPCAP on acute exacerbations of COPD, an important clinical and health-care problem.

**Added value of this study**

Although annual average concentrations of most pollutants have decreased from 2013 to 2017 in Beijing, significant positive associations with hospitalisations for acute exacerbations of COPD were still observed for both particulate and gaseous pollutants. However, the number of cases of acute exacerbation of COPD advanced by PM_2·5_ pollution exceeding the WHO 24-h PM_2.5_ target, as well as the associated health-care costs, was estimated to be reduced by nearly 42% from 2013 to 2017. To date, this study is the largest in China to investigate the associations between short-term air pollution exposures and acute exacerbations of COPD at a city level in a period when air quality is progressively improving. It is also the first study in China to show that implementation of a stringent air pollution control policy is beneficial for reducing COPD morbidity at the population level.

**Implications of all the available evidence**

In Beijing, the annual mean concentration of PM_2·5_ was reduced from 87 μg/m^3^ in 2013 to 58 μg/m^3^ in 2017. However, this 2017 concentration is still almost six times higher than the WHO target value of 10 μg/m^3^. Concentrations of some gaseous pollutants such as nitrogen dioxide and ozone remained stable over this period, indicating that controls against emissions of these pollutants need to be more strictly enforced, given that these pollutants also have adverse effects on respiratory health. Although our study highlights the positive health gains of APPCAP among patients with COPD, continuous monitoring of air quality and a long-term, multidimensional air pollution control policy are needed to safeguard public health and sustainable development in China.

In 2013, China launched the Air Pollution Prevention and Control Action Plan (APPCAP), in which various stringent measures^9^ (appendix p 3) were implemented nationwide to curb air pollution, particularly that from industrial sectors, to interim targets by the end of 2017.^10^ Beijing and the surrounding areas were among the most stringently targeted regions. During this 5-year period, concentrations of sulphur dioxide (SO_2_) were reduced by 70% and fine particulate matter (PM) by 33%.^10^ This progress provides an opportunity to study the potential health gains of this milestone policy.

In the current study, we investigated the associations between daily average concentrations of criteria air pollutants and daily hospitalisations for acute exacerbations of COPD in 2013–17 in Beijing. On the basis of the observed effect estimates, we calculated the number of cases of acute exacerbations of COPD advanced by air pollution each year to assess the potential effects of the recorded air-quality improvements.

## Methods

### Study setting and exposure data

Since 2013, PM_10_ (μg/m^3^), PM_2·5_ (μg/m^3^), SO_2_ (μg/m^3^), nitrogen dioxide (NO_2_; μg/m^3^), carbon monoxide (CO; mg/m^3^) and ozone (O_3_; μg/m^3^) have been routinely measured at 35 monitoring stations spread throughout Beijing (appendix p 2). The monitoring network is run by the Beijing Environmental Protection Bureau in accordance with the new Chinese national standard (GB 3095-2012). The monitoring stations were strategically assigned in representative locations to monitor emission sources from vehicles (road site, n=5), urban anthropogenic activities (urban site, n=23), natural activities (rural site, n=1), and regional transport or background (regional site on the outskirts of Greater Beijing, n=6). At each station, for each pollutant except O_3_, hourly data are usually available for at least 20 h each day to calculate the daily 24-h average (mean) concentration. For O_3_, hourly data should be available for at least 6 h in every 8 h to calculate a daily maximum 8-h moving average concentration.

A daily city-wide mean concentration for each pollutant, based on the daily mean readings from all these 35 stations, is reported on the Environmental Protection Bureau air-quality reporting platform. We obtained these daily city-wide average data from Jan 18, 2013, to Dec 31, 2017 (1809 days), and the quality check was satisfied (appendix p 4). Daily meteorological data (daily mean temperature [°C] and relative humidity [%]) were collected from the Beijing Meteorological Service website.

Of the 1809 days, there were 5 days with a missing city-wide daily average for all pollutants, which were excluded from the analysis. In the remaining 1804 days, a city-wide daily average was available for all pollutants, except 5 days of missing data and 97 days of distorted data for daily city-wide average PM_10_ (appendix p 4), and 34 days of missing daily city-wide average O_3_. To reconstruct the missing or distorted PM_10_ data for those 102 days, we used 24-h average concentrations for PM_2·5_ on the same date multiplied by the ratio between annual mean concentration for PM_10_ and annual mean concentration for PM_2·5_ derived for that year. The annual ratios derived were 1·312 for 2013, 1·444 for 2014, 1·441 for 2015, 1·379 for 2016, and 1·631 for 2017, which were similar to previously reported results.^11^ We did not reconstruct data on O_3_ because of the complex formation mechanism of this pollutant. Therefore, our analyses of PM_10_, PM_2·5_, PM_coarse_ (defined as PM >2·5–10 μm in diameter), SO_2_, NO_2_, and CO were based on the 1804-day dataset, and the analyses of O_3_ were based on a 1770-day dataset.

This study was approved by the Research Ethics Board of Beijing Chaoyang Hospital (approval number 2018-ke-303).

### Hospital data

Daily counts of hospital admissions for acute exacerbation of COPD were obtained from a hospital discharge database operated by Beijing Public Health Information Centre. In Beijing, each government and private hospital at secondary or tertiary level is required to submit their discharge records to the database.^12^ A three-tier health-care system is operated in China, where secondary and tertiary level hospitals are general hospitals eligible to provide specialised care.^13^ Each record includes data on age, sex, residential address, admitting hospital, date of admission, health-care cost, principal discharge diagnosis, and the corresponding International Classification of Diseases tenth revision (ICD-10) code following standard procedures. Using this information, we included patients with a primary discharge diagnosis of acute exacerbation of COPD (ICD-10 J44.0-J44.9), who were aged 18 years or older, and who were living in Beijing on a permanent basis. All admissions for acute exacerbation of COPD were from 119 hospitals (68 tertiary and 51 secondary).

### Statistical analysis

Daily hospitalisations for acute exacerbation of COPD, pollutant concentrations, and meteorological variables in 2013–17 were linked by date to allow a time-series analysis. We defined same-day exposure as lag0 and examined a priori daily exposure up to 4 days (single-day lag0 to lag4 and moving average of lag0–2 and lag0–4 concentrations) before hospitalisation, based on a systematic review.^6^ The associations between daily hospitalisations for acute exacerbation of COPD and average concentration of each pollutant were analysed with a generalised additive model estimating Poisson distribution, as follows:

log[E(Yt)]=intercept+βC-i+ps(calendar time,9)+ps(temp,3)+ps(RH,3)+public holiday+day of week

where E(Yt) represents the number of cases of acute exacerbation of COPD on day t; C is the city-averaged concentration; i is the day lag; β represents the log-relative risk (RR) of hospitalisation for acute exacerbation of COPD associated with a unit increase in each pollutant mean concentration; ps() indicates penalised spline function to filter out long-term trends and seasonal patterns in daily hospitalisations for acute exacerbation of COPD;^14^ temp is the daily mean temperature (°C); and RH is relative humidity (%). Public holiday and day of week were included as categorical variables. Degrees of freedom for calendar time, temperature, and relative humidity were selected based on the parameters used in previous studies.^15^, ^16^, ^17^

Apart from the single-pollutant models, we also investigated each association in two-pollutant models if Spearman correlation ratios between these pollutants were less than 0·7.^18^ Subgroup analyses at lag0 were done by age (18–64 years and ≥65 years), sex, and season (warm season [May to October] or cold season [November to April]). The Z test was used to compare the two estimates derived from each subgroup.

The smoothing function of the generalised additive model was used to graphically analyse the exposure–response relationships between the log-RR of hospitalisation for acute exacerbation of COPD and air pollutant concentrations at lag0.

For each single year from 2013 to 2017, we re-ran the main analyses for the associations between each air pollutant and acute exacerbations of COPD hospitalisation risk at lag0.

We did several sensitivity analyses by altering the generalised additive model: to exclude calendar time, as long-term trends and seasonal patterns might also be partly related to pollutant concentration; to replace calendar time with an interaction term of exposure-by-season; to increase the degrees of freedom of temperature and humidity to six; and to model moving averages for lag0–4 of temperature and humidity instead of the current day (lag0). The latter two analyses were to adjust potential non-linear and lagged confounding effects of weather conditions.^5^ Finally, we excluded the 102 days with reconstructed data for PM_10_ from the 1804-day dataset and re-ran the main analysis.

Using the following equation, we calculated the number of cases of acute exacerbations of COPD advanced by PM_2·5_, as an overall air-quality indicator, over the expected rates if daily concentrations had not exceeded the target in each year from 2013 to 2017:^12^

$$\sum_{t=1}^{365}\left(\frac{PMt-\mathrm{target}}{100}\times PARFlag0\times N\right)$$

where PMt was the city-wide average concentration of PM_2·5_ on day t; PARF represents the population-attributable risk fraction, calculated as (RR – 1) divided by RR, assuming the prevalence of air pollution exposure was 100%; and N is the daily mean number of cases in a particular year.

All statistical analyses were done in R (version 3.0.2) using the MGCV, DPLYR, and TTR packages. RRs of hospitalisation for acute exacerbation of COPD per IQR increase for each air pollutant were calculated.

### Role of the funding source

The funder of the study had no role in study design, data collection, data analysis, data interpretation, or writing of the report. The corresponding author had full access to all the data in the study and had final responsibility for the decision to submit for publication.

## Results

During 2013–17, the daily mean concentration of PM_10_ was 109·7 μg/m^3^ and of PM_2·5_ was 76·7 μg/m^3^ (table 1), both of which were considerably higher than the current Chinese grade I ambient air-quality standard (target 24-h mean concentrations 50 μg/m^3^ for PM_10_ and 35 μg/m^3^ for PM_2·5_) or WHO guidelines (target 50 μg/m^3^ for PM_10_ and 25 μg/m^3^ for PM_2·5_). 161 613 hospitalisations for acute exacerbation of COPD were recorded (mean 89 per day), with most patients being men and people aged 65 years or older.

**Table 1:** Air pollutant concentrations, weather conditions, and daily hospital admissions for acute exacerbation of chronic obstructive pulmonary disease in Beijing

	**Minimum**	**Maximum**	**Mean (SD)**	**Median (IQR)**
**Air pollutant concentrations**				
PM_10_, μg/m^3^	10·0	820·0	109·7 (79·1)	91·0 (54·0–140·0)
PM_2·5_, μg/m^3^	5·0	467·0	76·7 (66·7)	58·0 (29·0–101·0)
PM_coarse_, μg/m^3^	0·0	461·0	33·0 (29·1)	27·0 (16·0–41·0)
NO_2_, μg/m^3^	8·0	155·0	50·5 (24·2)	44·0 (33·0–63·0)
SO_2_, μg/m^3^	2·0	139·0	15·1 (18·4)	8·0 (4·0–19·0)
O_3_, μg/m^3^	2·0	292·0	95·8 (62·2)	83·0 (50·0–135·0)
CO, mg/m^3^	0·2	8·0	1·2 (1·0)	0·9 (0·6–1·4)
**Meteorological measures**				
Temperature, °C	−16·0	32·0	13·1 (11·0)	14·0 (2·0–23·0)
Relative humidity, %	8·0	97·0	53·2 (20·1)	53·0 (38·0–69·5)
**Hospital admissions (number of cases per day)**				
Total	17	220	89 (36)	89 (60–113)
Female	2	90	29 (14)	27 (18–37)
Male	9	153	60 (23)	62 (39–76)
Age <65 years	0	43	14 (7)	14 (9–19)
Age ≥65 years	13	184	75 (30)	75 (51–94)
Warm season, May to October	17	168	80 (30)	82 (52–102)
Cool season, November to April	19	220	99 (38)	99 (68–126)

Data are for 1804 days (or 1770 days for O_3_) from 2013 to 2017. Air pollutant concentrations are 24-h averages, except for O_3_ concentrations, which are 8-h averages. PM=particulate matter. NO_2_=nitrogen dioxide. SO_2_=sulphur dioxide. O_3_=ozone. CO=carbon monoxide.

**Figure 1:**
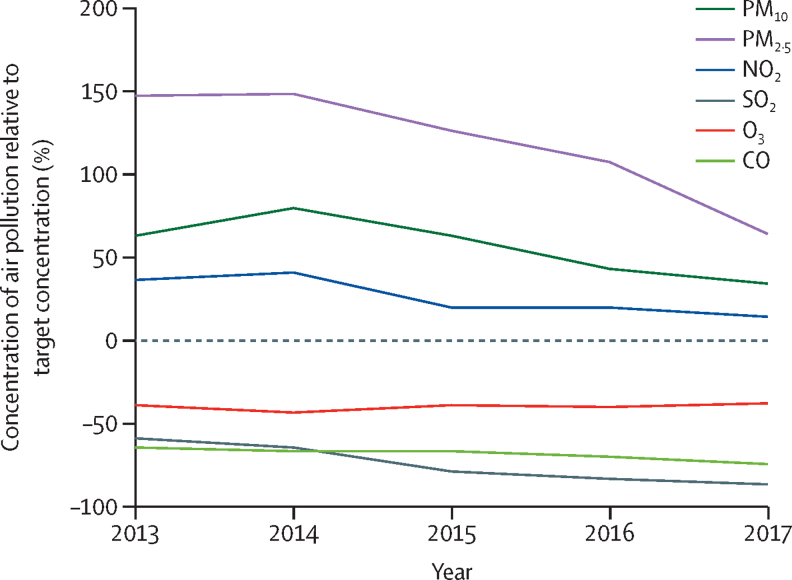
Annual mean average concentrations of the six criteria air pollutants in Beijing in 2013–17 as percentages of the Chinese grade II target annual concentrations The dashed line denotes the Chinese grade II target annual concentration. Values are the percentage increase or decrease of each concentration relative to the target concentration (0%). CO=carbon monoxide. NO_2_=nitrogen dioxide. O_3_=ozone. PM=particulate matter. SO_2_=sulphur dioxide.

Over the 5-year period studied, annual mean SO_2_ concentration decreased by 68% (from 24·5 μg/m^3^ [SD 23·2] in 2013 to 7·7 μg/m^3^ [8·4] in 2017) and PM_2·5_ concentration by 33% (from 86·8 μg/m^3^ [65·8] to 57·7 μg/m^3^ [55·5]), whereas the concentration of O_3_ remained stable (figure 1; appendix p 5). Concentrations of PM_10_, PM_2·5_, NO_2_, and CO were lowest in summer (June to August) and highest in winter (November to February) (appendix p 6), but the opposite trend was observed for O_3_. Concentrations of PM_coarse_ were consistently higher in spring (March to May). PM_10_, PM_2·5_, NO_2_, and CO concentrations showed strong positive correlations with one another (Spearman's r>0·7), whereas PM_coarse_ and PM_2·5_ showed a weak positive correlation (r=0·247; appendix p 9). SO_2_ concentration showed moderate positive correlations with concentrations of PM_10_, PM_2·5_, and NO_2_, and CO (r>0·4 to <0·7).

**Figure 2:**
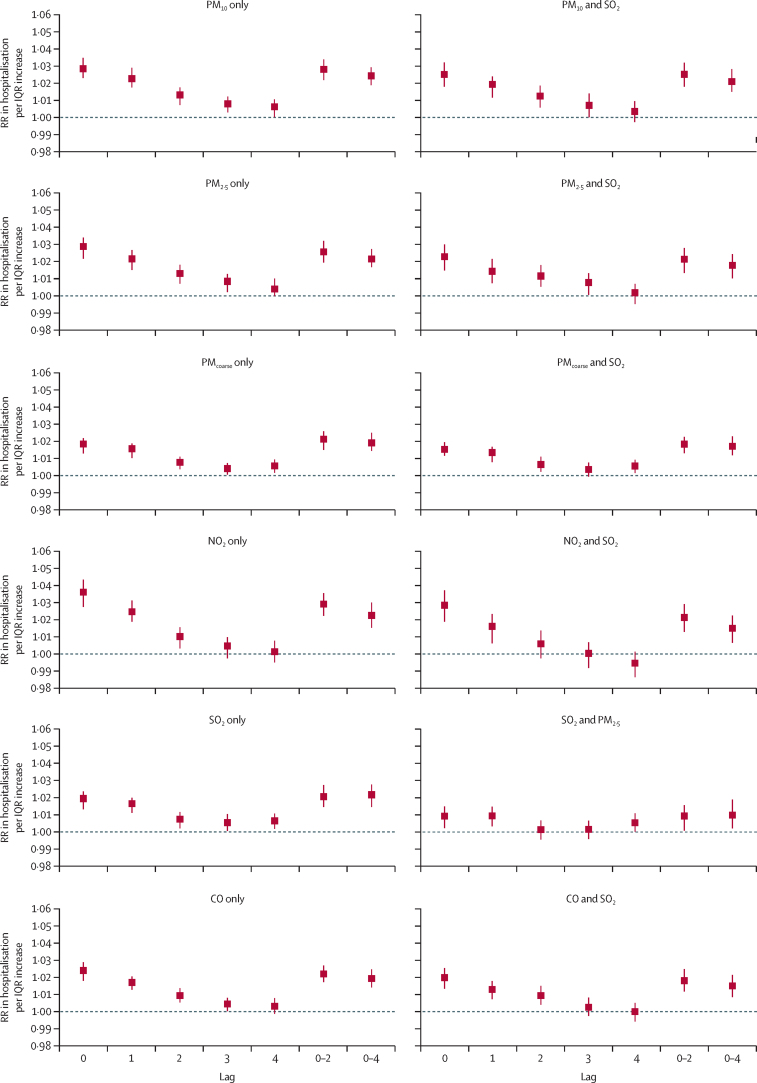
RR of hospitalisation for acute exacerbation of COPD associated with pollutants in single-pollutant and two-pollutant models at different lag days during 2013–17 Data are RR (95% CI) per IQR increment of pollutant concentration. CO=carbon monoxide. NO_2_=nitrogen dioxide. O_3_=ozone. PM=particulate matter. RR=relative risk. SO_2_=sulphur dioxide.

In single-pollutant models at lag0, the RR of hospitalisation for acute exacerbation of COPD per IQR increase in pollutant was 1·029 (95% CI 1·023–1·035) for PM_10_, 1·028 (1·021–1·034) for PM_2·5_, 1·018 (1·013–1·022) for PM_coarse_, 1·036 (1·028–1·044) for NO_2_, 1·019 (1·013–1·024) for SO_2_, and 1·024 (1·018–1·029) for CO (figure 2; appendix pp 10–12). All these effect estimates were highest at lag0 and showed a decreasing trend to lag4. These results did not substantially change in most of the two-pollutant models, except that the associations with PM_coarse_ or SO_2_ became non-significant when PM_10_ was further adjusted (appendix pp 14–17). Associations with moving-day average (lag0–2 and lag0–4) exposures were significant in both single-pollutant and two-pollutant models for all pollutants, with effect estimates similar to those seen at lag0.

In the warm season, increased O_3_ exposures at lag0 and lag0–2 were significantly associated with increased hospitalisations for acute exacerbations of COPD (table 2), for which the association persisted after further adjustment for NO_2_ or CO, but not for PM (except PM_coarse_ at lag0). In the cold season at lag0, O_3_ was significantly associated with decreased hospitalisations for acute exacerbation of COPD in all models, as was O_3_ at lag0–2 and lag0–4, except in the model also adjusted for CO.

**Table 2:** Associations between daily average concentration of O_3_ (per IQR of 85 μg/m^3^ higher) and daily hospital admissions for acute exacerbation of chronic obstructive pulmonary disease in Beijing (2013–17, 1770 days) in single-pollutant and two-pollutant models

	**O_3_ only**	**O_3_ and PM_10_**	**O_3_ and PM_coarse_**	**O_3_ and PM_2·5_**	**O_3_ and NO_2_**	**O_3_ and CO**	**O_3_ and SO_2_**
**Warm season (May to October)**							
Lag0	1·027 (1·010–1·044)*	1·008 (0·991–1·025)	1·022 (1·005–1·039)*	1·006 (0·989–1·023)	1·025 (1·008–1·042)*	1·025 (1·008–1·042)*	1·018 (1·001–1·035)*
Lag1	1·019 (1·004–1·035)*	1·009 (0·994–1·025)	1·017 (1·001–1·033)*	1·011 (0·996–1·027)	1·019 (1·004–1·035)*	1·019 (1·004–1·034)*	1·015 (0·999–1·031)
Lag2	1·004 (0·990–1·018)	0·996 (0·982–1·011)	1·002 (0·988–1·016)	0·997 (0·983–1·012)	1·004 (0·989–1·018)	1·003 (0·989–1·018)	1·003 (0·988–1·019)
Lag3	1·000 (0·986–1·014)	0·994 (0·979–1·008)	0·998 (0·984–1·013)	0·994 (0·980–1·009)	0·999 (0·985–1·013)	1·000 (0·986–1·014)	1·002 (0·987–1·017)
Lag4	0·995 (0·982–1·009)	0·991 (0·977–1·006)	0·993 (0·980–1·007)	0·993 (0·978–1·007)	0·994 (0·980–1·008)	0·995 (0·981–1·009)	0·991 (0·976–1·005)
Lag0–2	1·027 (1·007–1·048)*	1·006 (0·985–1·027)	1·019 (0·998–1·040)	1·010 (0·989–1·030)	1·028 (1·008–1·048)*	1·026 (1·006–1·047)*	1·016 (0·995–1·038)
Lag0–4	1·019 (0·996–1·042)	1·001 (0·978–1·025)	1·011 (0·988–1·034)	1·006 (0·983–1·029)	1·023 (1·000–1·047)	1·019 (0·997–1·043)	1·007 (0·983–1·032)
**Cool season (November to April)**							
Lag0	0·952 (0·936–0·969)*	0·957 (0·941–0·974)*	0·960 (0·943–0·977)*	0·955 (0·938–0·971)*	0·964 (0·947–0·982)*	0·966 (0·949–0·984)*	0·955 (0·938–0·972)*
Lag1	0·970 (0·956–0·985)*	0·982 (0·967–0·998)*	0·975 (0·960–0·989)*	0·979 (0·963–0·995)*	0·984 (0·967–1·002)	0·989 (0·973–1·007)	0·975 (0·960–0·991)*
Lag2	0·991 (0·977–1·005)	0·997 (0·982–1·012)	0·992 (0·978–1·006)	0·996 (0·981–1·011)	0·993 (0·977–1·009)	1·001 (0·985–1·018)	0·989 (0·975–1·004)
Lag3	0·988 (0·974–1·001)	0·989 (0·975–1·004)	0·988 (0·975–1·002)	0·988 (0·974–1·003)	0·980 (0·965–0·996)*	0·989 (0·973–1·005)	0·986 (0·972–1·001)
Lag4	0·988 (0·975–1·002)	0·990 (0·976–1·005)	0·989 (0·975–1·003)	0·988 (0·973–1·003)	0·979 (0·963–0·995)*	0·988 (0·972–1·005)	0·986 (0·972–1·001)
Lag0–2	0·956 (0·937–0·976)*	0·969 (0·948–0·990)*	0·963 (0·943–0·983)*	0·964 (0·943–0·985)*	0·970 (0·948–0·992)*	0·979 (0·957–1·002)	0·959 (0·939–0·979)*
Lag0–4	0·960 (0·939–0·981)*	0·972 (0·950–0·994)*	0·963 (0·942–0·984)*	0·967 (0·945–0·989)*	0·963 (0·940–0·987)*	0·980 (0·955–1·005)	0·961 (0·940–0·982)*

Data are relative risk (95% CI). O_3_=ozone. PM=particulate matter. NO_2_=nitrogen dioxide. CO=carbon monoxide. SO_2_=sulphur dioxide.

* Statistically significant (p<0·05).

At lag0, for all pollutants except PM_coarse_ and O_3_, larger effect estimates were seen in women than in men, and in people aged 65 years or older than in those younger than 65 years (Z test p<0·05; appendix p 7). Effect estimates for PM_2·5_ were significantly higher in the warm season than in the cold season.

**Figure 3:**
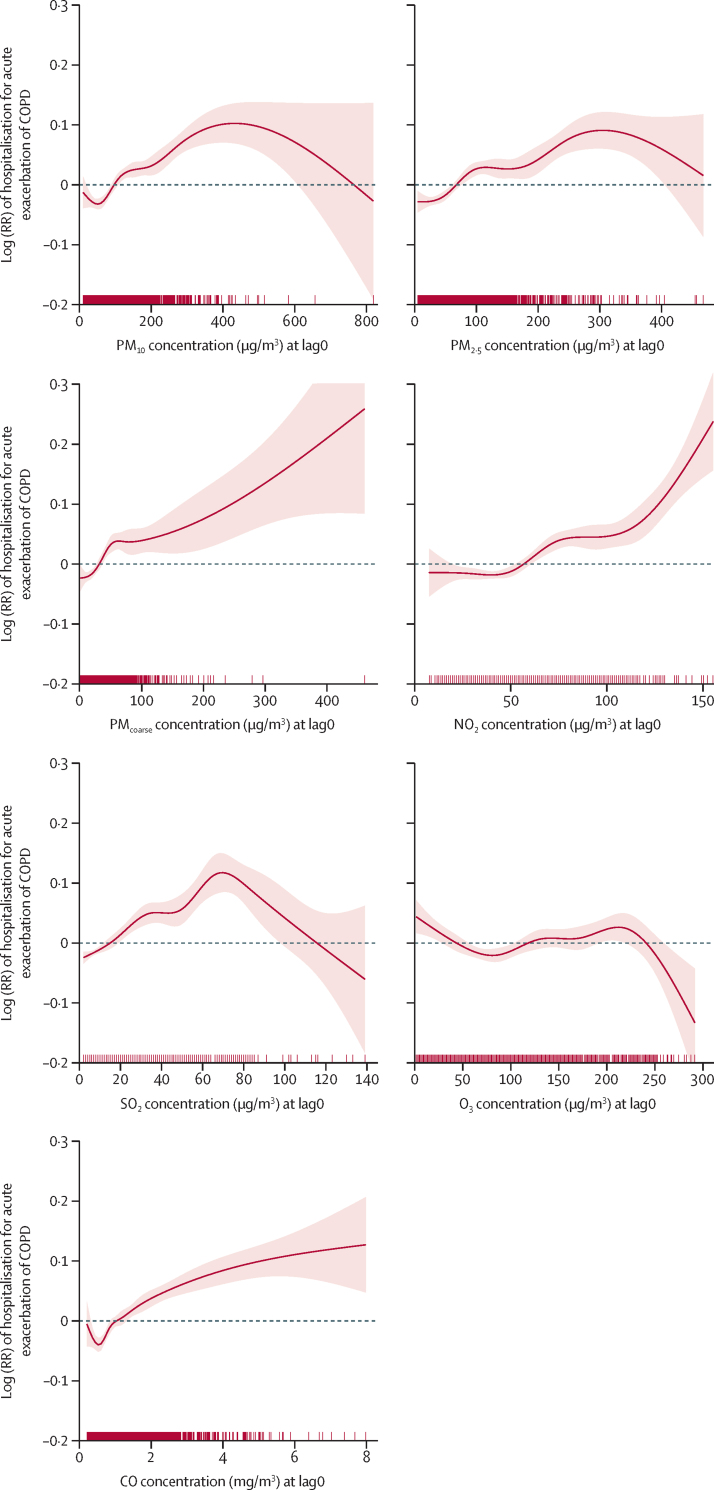
Exposure–response relationships between each pollutant and hospitalisation for acute exacerbation of COPD in single-pollutant models at lag0 during 2013–17 Red tick marks along the x-axes represent individual observations. CO=carbon monoxide. COPD=chronic obstructive pulmonary disease. NO_2_=nitrogen dioxide. O_3_=ozone. PM=particulate matter. RR=relative risk. SO_2_=sulphur dioxide.

Positive exposure–response curves at lag0 were observed at least up to 400 μg/m^3^ for PM_10_ and up to 300 μg/m^3^ for PM_2·5_, whereas no saturation effect was observed for PM_coarse_ even up to 400 μg/m^3^ (figure 3). Various patterns were observed for the gaseous pollutants (figure 3).

The yearly coefficients at lag0 for most pollutants fluctuated throughout 2013–17 (appendix p 8).

Results from all sensitivity analyses were generally in line with the main results (figure 2). When the time variable was excluded (appendix p 18) or replaced with an exposure-by-season interaction term (appendix p 19), all significant associations persisted but the effect estimates increased. When the degrees of freedom of weather conditions were increased (appendix p 20), the results remained very similar to those of the main analysis (figure 2). Additionally, when the moving average of lag effects of weather conditions was modelled (appendix p 21), effect estimates were slightly reduced but remained significant. Results also did not change substantially after excluding days with missing or distorted PM_10_ data (appendix p 22).

The number of days that city-averaged PM_2·5_ concentration exceeded the WHO 24-h target (25 μg/m^3^) was reduced from 298 in 2013 to 256 in 2017 (table 3). There was a decreasing trend in the number of cases of acute exacerbations of COPD advanced by PM_2·5_ pollution above the expected rates if daily concentrations had not exceeded either the Chinese or WHO targets (table 3). Based on the WHO 24-h target, the number of acute exacerbations of COPD cases advanced by PM_2·5_ was 12 679 in 2013 and 7377 in 2017, corresponding to a decrease of nearly 42% between those two timepoints. A similar percentage reduction was observed for health-care cost (table 3).

**Table 3:** Number of cases of acute exacerbation of COPD advanced by PM_2·5_ pollution above the expected rates if daily PM_2·5_concentrations had not exceeded the standard 24-h targets each year

	**2013**	**2014**	**2015**	**2016**	**2017**
**Chinese grade II 24-h target (75 μg/m^3^)**					
Number of days target not attained	153	163	142	132	85
Number of cases	6095	4269	5042	1851	2714
Health-care cost*, million ¥	108·4	76·6	93·2	34·5	48·2
**Chinese grade I 24-h target (35 μg/m^3^)**					
Number of days target not attained	275	270	255	240	212
Number of cases	11 030	7487	8876	3524	6020
Health-care cost*, million ¥	196·2	134·4	164·1	65·6	107·0
**WHO 24-h target (25 μg/m^3^)**					
Number of days target not attained	298	301	287	281	256
Number of cases	12 679	8529	10 237	4122	7377
Health-care cost*, million ¥	225·5	153·1	189·3	76·7	131·1

COPD=chronic obstructive pulmonary disease. ¥=Chinese yuan.

* Number of estimated cases multiplied by mean average health-care cost of each case of acute exacerbation of COPD in that year in Beijing. Average health-care cost (inflation-adjusted) for each case of acute exacerbation of COPD each year was extracted from the same hospital discharge database operated by Beijing Public Health Information Centre. The average health-care cost for each case of acute exacerbation of COPD in Beijing was ¥17 790·2 in 2013, ¥17 948·0 in 2014, ¥18 489·4 in 2015, ¥18 616·4 in 2016, and ¥17 778·8 in 2017.

## Discussion

We found significant associations between short-term exposures to air pollution and hospitalisations for acute exacerbation of COPD, with RRs at lag0 ranging from 1·018 to 1·036 for each IQR increase in concentration. By lag4, most of these increased risks became non-significant. Effects of moving-day average exposures (lag0–2 and lag0–4) were also statistically significant and similar to those seen at lag0. Women and patients aged 65 years or older were most susceptible. The shapes of each exposure–response relationship varied greatly by type of pollutant in our study, which probably reflects the variations in biological mechanisms and characteristics, including toxicity, of each pollutant.

Our effect estimates expressed per 10 μg/m^3^ increase (appendix p 13) were generally lower, especially for PM, than those in other Asian studies done at least a decade ago (appendix p 24). Although the study populations and sources of PM_2·5_ data were not directly comparable, our effect estimate per 10 μg/m^3^ increase of PM_2·5_ at lag0 (1·004 [95% CI 1·003–1·005]; appendix p 25) was lower than those reported in previous studies of Beijing residents conducted in 2010–12 (1·007 [1·006–1·007])^16^ and in 2013 (1·015 ([1·001–1·028]).^19^

APPCAP has mainly targeted heavy industries and has resulted in an appreciable reduction in concentrations of SO_2_ and PM_2·5_ in Beijing. It is likely that the compositions of air pollutant mixtures will have changed (eg, lower sulphur compositions) over the years, and that these changes could have positive effects on health. A 2018 study of 74 cities in China estimated substantial reductions in mortality as an effect of APPCAP.^9^ This finding is further supported by our estimation of decreased numbers of cases of acute exacerbations of COPD advanced by PM_2·5_ pollution, highlighting the effectiveness of such air pollution control policy in reducing respiratory morbidity. However, lasting health benefits from improved air quality remain to be confirmed. A long-term, multidimensional air pollution control strategy is needed in China to safeguard public health and reduce health-care costs.

It has been hypothesised that air pollutants could induce airway epithelial damage, inhibit mucociliary clearance, and impair macrophage function through activation of inflammatory cells and their mediators as well as through promotion of intracellular oxidative stress.^20^ These pathways might either directly trigger an exacerbation, or collectively create a pulmonary microenvironment with impaired immune function that makes the host more susceptible to viral and bacterial infections—the major causes of acute exacerbations of COPD. Besides the physical and chemical compositions of pollutants that might, in part, underlie the mechanisms leading to acute exacerbations of COPD, some airborne microbes (eg, pathogenic bacteria and fungi) might also play a role.^21^

Relatively few studies^22^ have investigated the short-term effects of PM_coarse_ on acute exacerbations of COPD. In our study, the exposure–response curve followed a non-linear pattern, being steeper at lower concentrations but shallower at higher concentrations, and no saturation effect was evident. This finding indicates that PM_coarse_ (or, similarly, PM_2·5_), even at a relatively low concentration, could increase risk of acute exacerbation events in COPD, although the threshold for so-called safe concentrations remains to be established. Previous studies have reported that PM_coarse_ has short-term health effects at least as strong as those of PM_2·5_, but the effects of PM_coarse_ were generally higher for respiratory than for cardiovascular outcomes because of the physical and chemical differences in these particles.^22^ Unlike PM_2·5_ which can travel deep into the respiratory system to the alveoli and terminal bronchioles, and even cross the air–blood barrier, PM_coarse_ is mainly deposited in the primary bronchi. PM_coarse_ is formed of more visible forms of PM, including road dust, soil, and black smoke.^23^ In epidemiological studies, PM_coarse_, but not PM_2·5_, was associated with an increased prevalence of respiratory symptoms,^24^, ^25^ indicating that PM_coarse_ might have a greater role in the triggering of acute exacerbations of COPD than does PM_2·5_. Beijing is often affected by dust storms with extremely high concentrations of coarse particles, and studies have suggested a link between these dust storms and hospitalisations for respiratory disease.^26^, ^27^ The significance of our findings for PM_coarse_ appeared unaffected by adjustment for PM_2·5_, but epidemiological and toxicological evidence of the effects of coarse particles on respiratory outcomes warrants further investigation.

Very few studies from Asia have explored the acute effects of CO and the risk of acute exacerbations of COPD. In our study, we observed a 3% increase in acute exacerbations of COPD per 1 mg/m^3^ increase in CO at lag0 (appendix p 13), consistent with pooled estimates reported for Europe (4%) and North America (2%).^6^ However, two studies in Shanghai^28^ and Hong Kong^29^ have shown that low ambient concentrations of CO are protective against COPD exacerbation, even after co-adjusting for other traffic-related pollutants. Both studies cautiously suggested that this link is possible because low concentrations of CO can have anti-inflammatory and antimicrobial effects, as reported in both experimental and human studies.^28^, ^30^, ^31^ Our study had similarly low concentrations of CO, but the correlations between CO and other traffic-related pollutants (PM_2·5_ and NO_2_) in our study were high, which precluded co-adjustments with these pollutants.

Ambient concentrations of SO_2_ in China have decreased markedly since 2013,^9^, ^32^ as supported by our data, mainly because of strict control measures among industries and a steady structural change in energy consumption. Despite this reduction, over this 5-year period, we found a modest positive association between SO_2_ and hospitalisations for acute exacerbations of COPD, even after adjusting for PM_2·5_. The significant association between exacerbations and SO_2_ observed in 2013 seem to diminish over the years to 2017, a pattern that was not seen for other pollutants. However, this finding needs cautious interpretation until air pollution concentrations for future years become available to allow the effects of long-term air-quality improvement on health gains to be studied.

The concentrations of O_3_ remained stable from 2013–17. Background O_3_ concentrations might remain relatively constant for many years in urban areas in China, and even increase if measures against nitrogen oxides (NO_X_) emissions are adopted rigorously, as shown in Europe and North America.^33^ The Global Burden of Disease study estimated that about 254 000 deaths from COPD in 2015 were attributable to O_3_.^34^ Given this context, and that our results for O_3_ during the warm season were robust to adjustments for some other gaseous pollutants, continuous monitoring and mitigation measures for O_3_ are needed.

The seasonal effects of O_3_ on acute exacerbations of COPD remain unclear, as we observed positive associations in the warm season but negative associations in the cold season. In Beijing, concentrations of O_3_ during the warm season are high, and people tend to go outdoors and open windows more often; in the cold season, concentrations are low, while people mostly stay indoors and ventilation is reduced because of heating. These differential behaviours of individuals could also partly explain the higher effect estimates for PM_10_ and PM_2·5_ in the warm season. Additionally, high temperatures might have a synergistic role.^35^ As with two other studies of Beijing residents,^16^, ^19^ we found that women with COPD were more susceptible to acute air pollution effects. Patients aged 65 years and older were also susceptible because they are likely to have a compromised immune system, and the health of patients with COPD generally deteriorates rapidly after 65 years of age.

As with many previous studies, we have only considered the temporal variations of the effects of air pollutants on hospitalisations for acute exacerbations of COPD. However, spatial variations of these health effects should not be disregarded, especially in megacities such as Beijing, where spatial variation in concentrations of air pollutants could vary greatly. The southern part of Greater Beijing reportedly has worse air quality than that in the northern part, while monitoring stations near traffic or in the city centre have (as expected) the highest concentrations of some pollutants.^36^ In addition, confounding factors operate at the area level across the city, which might also affect the spatial variations of the health effects. For example, a study in Beijing reported that, despite the lower air pollution concentration in suburban and rural areas compared with urban areas, cardiovascular mortality risk in relation to air pollution was higher in suburban and rural areas than in urban areas.^37^ Although different compositions of air pollutants across the areas probably contribute to this effect, factors such as access to quality health care, age structure of populations, and availability of protection measures also contribute. Beyond the scope of this study, a separate, careful investigation involving the collection of station-specific air quality data and contextual area-level data would be needed to understand the spatial variations of acute exacerbations of COPD risks in Beijing.

This is by far the largest time-series study in China to study the short-term effects of air pollution on hospitalisations for acute exacerbations of COPD among the whole population of Beijing at a time when various measures were being implemented to reduce air pollution. Our study had some limitations. First, because this was an ecological analysis in which individual-level confounding factors were not considered, modelled estimates should not be interpreted as predictive of individual hospitalisation probability. Some factors are unlikely to change over a short period, but time-varying factors such as seasonal viral or bacterial infection patterns might confound the studied relationships. Second, we only had data on daily, outdoor, city-wide average concentrations of each air pollutant, which we correlated with daily hospitalisations for acute exacerbation of COPD in the whole of Beijing. This method will have introduced bias to health estimates because place of residence and time-activity (eg, time spent indoors, including in the home and workplace, and the associated exposures to indoor sources) were not taken into account. People spend most of their time indoors, and outdoor pollution does penetrate indoors but with variable infiltration efficiency. This exposure misclassification is likely to underestimate, although not necessarily invalidate, the effect estimate.^38^, ^39^ Furthermore, assigning estimates from the nearest monitoring station to residence did not necessarily improve the correlation between personal exposures and ambient concentrations.^40^ Ongoing studies^41^ using personal air pollution monitoring will be useful to bridge this gap. Third, our study outcome was confirmed hospitalisation for acute exacerbation of COPD, which might only represent the most severe cases. Air pollution can also have specific effects on different types of exacerbations,^42^ and these effects should be investigated further if data became available. Fourth, as the air pollution concentration in Beijing was in decline over the study years, there might have been fewer deaths among patients with COPD^9^ and thus the existing COPD populations might have increased in size, which could have affected our estimates. Finally, other than effective emission control measures, trends in economic development, meteorological conditions, and health care might all contribute to air quality and health management,^43^ and these contributions should be carefully considered in future investigations.

In conclusion, acute exposures to both particulate and gaseous pollutants were significantly associated with hospitalisations for acute exacerbation of COPD in Beijing. Although the APPCAP has shown positive effects in terms of reducing air pollution and COPD morbidity in our study population, the concentrations of ambient air pollution are still dangerously high, warranting continued collective mitigation measures to achieve substantial health benefits.

For the **Environmental Protection Bureau air-quality reporting platform** see http://zx.bjmemc.com.cn/

For the **Beijing Meteorological Service** see http://www.bjmb.gov.cn/
