# Supplementary material for: Associations between daily air quality and hospitalisations for acute exacerbation of chronic obstructive pulmonary disease in Beijing, 2013–17: an ecological analysis
Source: Lancet Planet Health. 2019 Jun;3(6):e270–9. doi: 10.1016/S2542-5196(19)30085-3 (PMC6610933; doi:10.1016/S2542-5196(19)30085-3)
Supplement: Supplementary appendix [file mmc1.pdf]

THE LANCET
Planetary Health

Supplementary appendix

This appendix formed part of the original submission and has been peer reviewed. We post it as supplied by the authors.

Supplement to: Liang L, Cai Y, Barratt B, et al. Associations between daily air quality and hospitalisations for acute exacerbation of chronic obstructive pulmonary disease in Beijing, 2013–17: an ecological analysis. *Lancet Planet Health* 2019: **3:** e270–79.

Figure S1: Air quality monitoring stations around Beijing

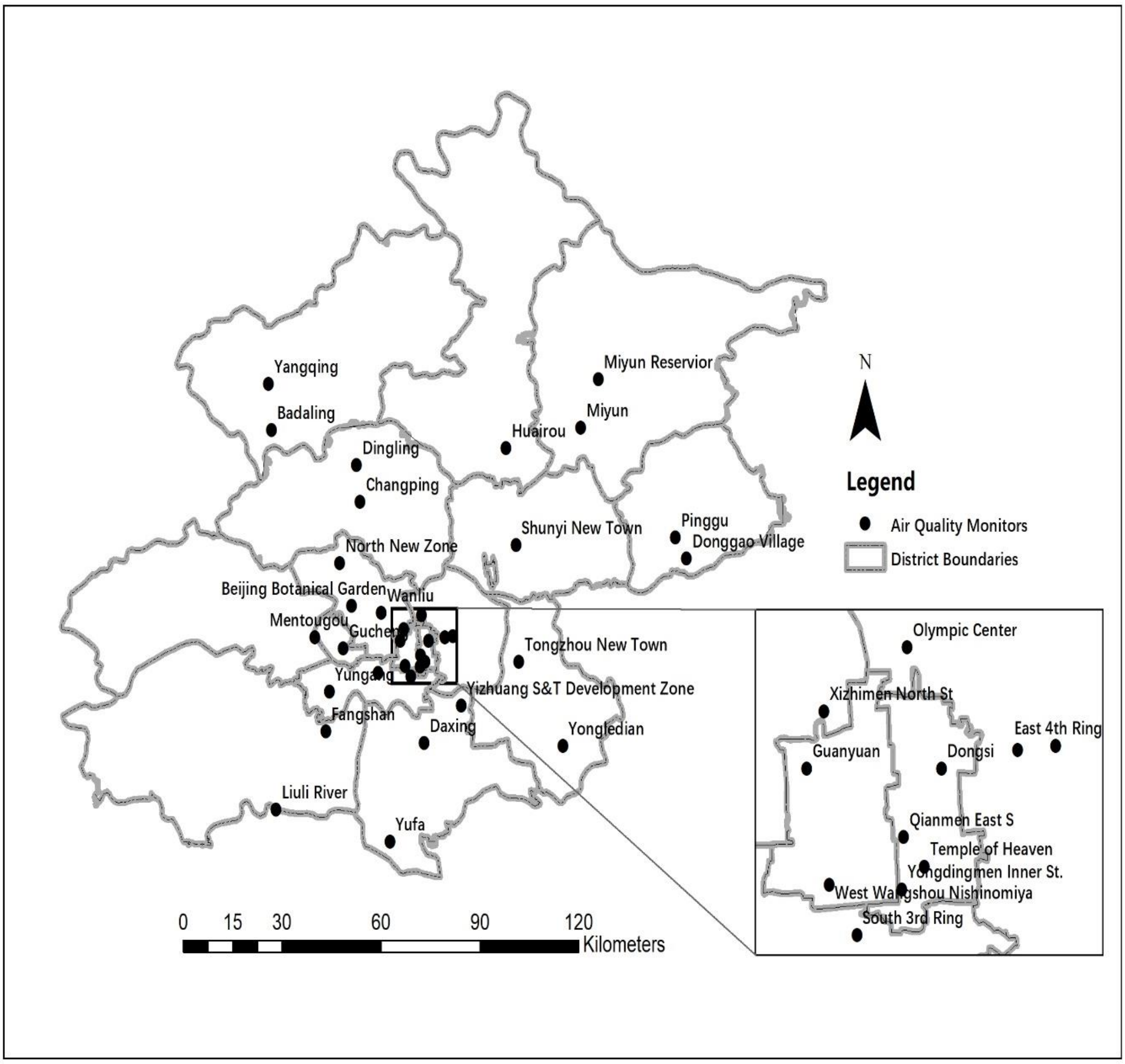

Supplementary text-1

Ten measures of China's Air Pollution Prevention and Control Action Plan (2013-2017), as referenced from *Huang et al Lancet Planet Health. 2018 Jul;2(7):e313-e323.*

1 Increase efforts towards comprehensive control and reduce emissions of multi-pollutants

2 Optimise the industrial infrastructure and promote industrial restructuring

3 Accelerate technology transformation and improve innovation capability

4 Adjust the energy infrastructure and increase clean energy supply

5 Strengthen environmental thresholds and optimise industrial layout

6 Promote the role of market mechanisms and improve environmental economic policies

7 Improve legal and regulatory systems and continue supervision and management based on law

8 Establish regional coordination mechanisms and integrated environmental management

9 Establish monitoring and warning systems to cope with air pollution episodes

10 Clarify the responsibilities of the government, enterprises, and society, and mobilise public participation

Supplementary text-2

Since we directly obtained the city-wide daily average air pollution data from the EPB air quality reporting platform (http://zx.bjmemc.com.cn/), we did some further quality checks on these data.

1. We obtained the hourly data from all 35 stations for $PM_{2.5}$ and PM_{10} each day during our study period (18-January-2013 to 31-December 2017).
2. We calculated the station-specific daily average concentration for $PM_{2.5}$ and PM_{10}, when there were at least 20 valid hourly observations in a day.
3. We then calculated the city-wide daily average concentrations, which were based on at least 12 stations with valid station-specific daily average data
4. We then compared the city-wide daily average concentration for $PM_{2.5}$ and PM_{10} that we calculated in Step 3, against the data we directly obtained from the EPB website. As in the figure below, there was a very good agreement between the two datasets.

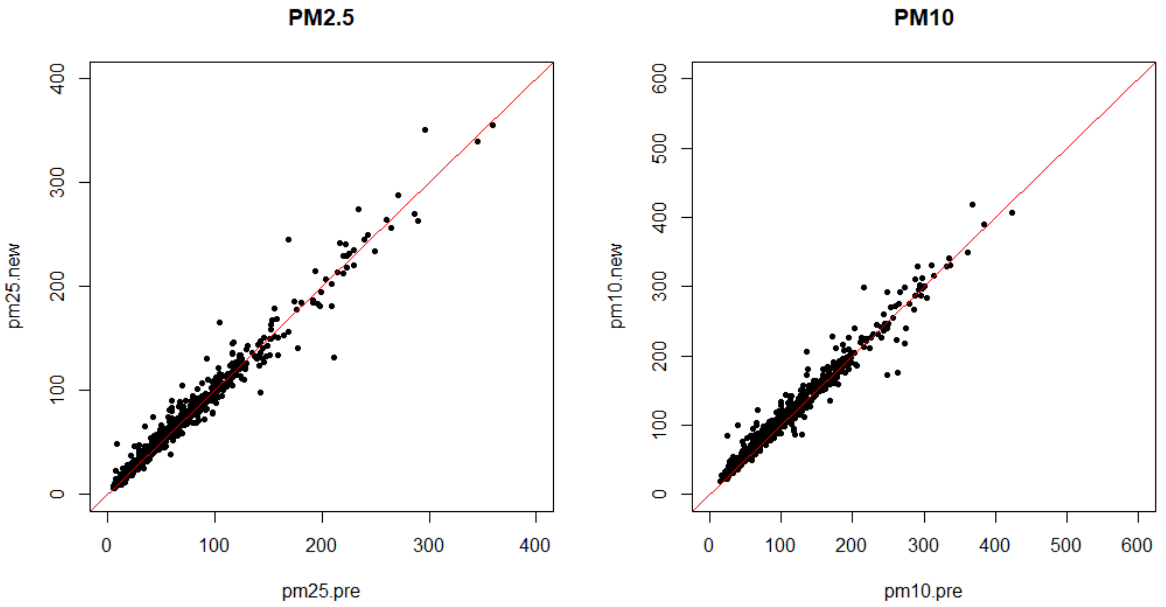

We identified 97 days of distorted data for daily city-wide average PM_{10}, as its reading was less than that of $PM_{2.5}$ on the same date. We deemed that data for daily $PM_{2.5}$ was correct, as we compared our data obtained from the EPB website with those from the monitoring sites in Beijing operating by the State air quality monitoring network which shows a very good agreement.

Supplemental Table 1 Annual changes in air pollutants, meteorological factors and daily hospital admissions for acute exacerbation of chronic obstructive pulmonary disease (AECOPD) in Beijing (2013-2017, n = 1,804 days (1770 days for O_3)).

		2013		2014		2015		2016		2017	
		Mean	SD	Mean	SD	Mean	SD	Mean	SD	Mean	SD
Air pollutant concentrations*	PM_{10} (μg/m^3)	113·9	70·5	126·1	83·4	114·1	89·2	100·3	71·6	94·1	75·2
	$PM_{2·5}$(μg/m^3)	86·8	65·8	87·3	72·5	79·2	70·8	72·7	63·6	57·7	55·5
	PM_{coarse}(μg/m^3)	27·1	20·2	38·8	29·2	34·9	35·8	27·6	23·5	36·4	31·8
	NO_2 (μg/m^3)	54·4	24·0	56·5	25·9	47·9	24·1	48·1	24·2	45·7	20·8
	SO_2 (μg/m^3)	24·5	23·2	21·2	24·2	12·6	13·8	10·0	10·3	7·7	8·4
	O_3 (μg/m^3)	98·1	57·1	89·4	59·5	97·9	65·5	95·1	64·9	98·5	62·8
	CO (mg/m^3)	1·4	1·0	1·3	0·9	1·3	1·1	1·2	1·0	1·0	0·8
Meteorological measures	Temperature (℃)	13·1	10·8	13·4	10·9	13·1	10·9	12·9	11·5	13·2	11·2
	Relative humidity (%)	52·9	20·0	50·8	19·1	56·2	20·5	53·8	19·6	52·4	21·0
Hospital admission (cases per day)	Total	102	37	112	40	75	26	81	29	78	29
	Male	64	24	74	26	52	18	57	20	56	21
	Female	38	14	39	16	23	10	24	10	22	10
	<65years	16	8	20	8	13	5	13	6	12	6
	≥65years	85	31	93	33	62	23	68	25	66	25
	Cool season	113	39	127	40	83	28	92	31	82	31
	Warm season	91	32	98	33	66	22	71	23	74	27

SD: standard deviation· Cool season: from November to April; Warm season: from May to October· *presented as 24-hour average concentration, except for ozone which was presented as 8-hour average concentration·

Figure S2 Monthly average concentration of air pollutants from 2013 to 2017 in Beijing

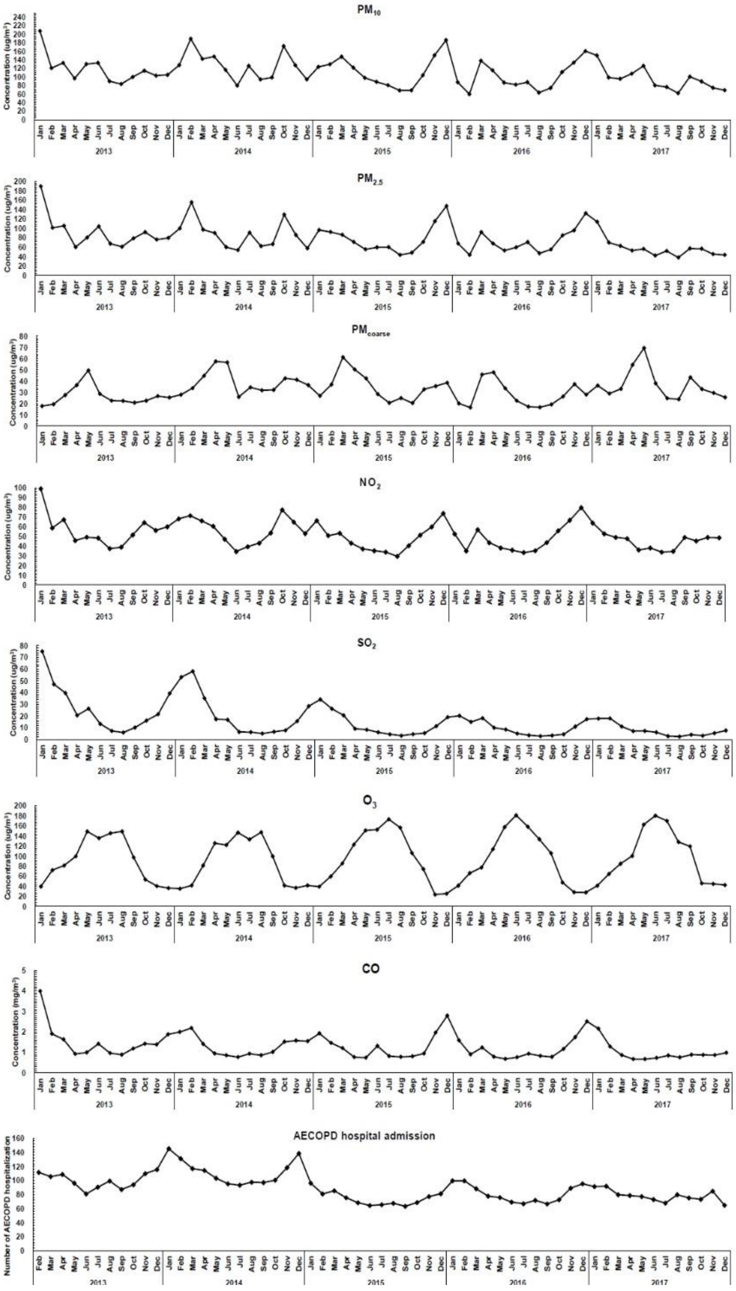

Figure S3 **Relative risk and 95% CI per interquartile-range increment for each pollutant in AECOPD hospital admissions in single-pollutant models atLag0 during 2013-2017: analysis by sex, age and season.**

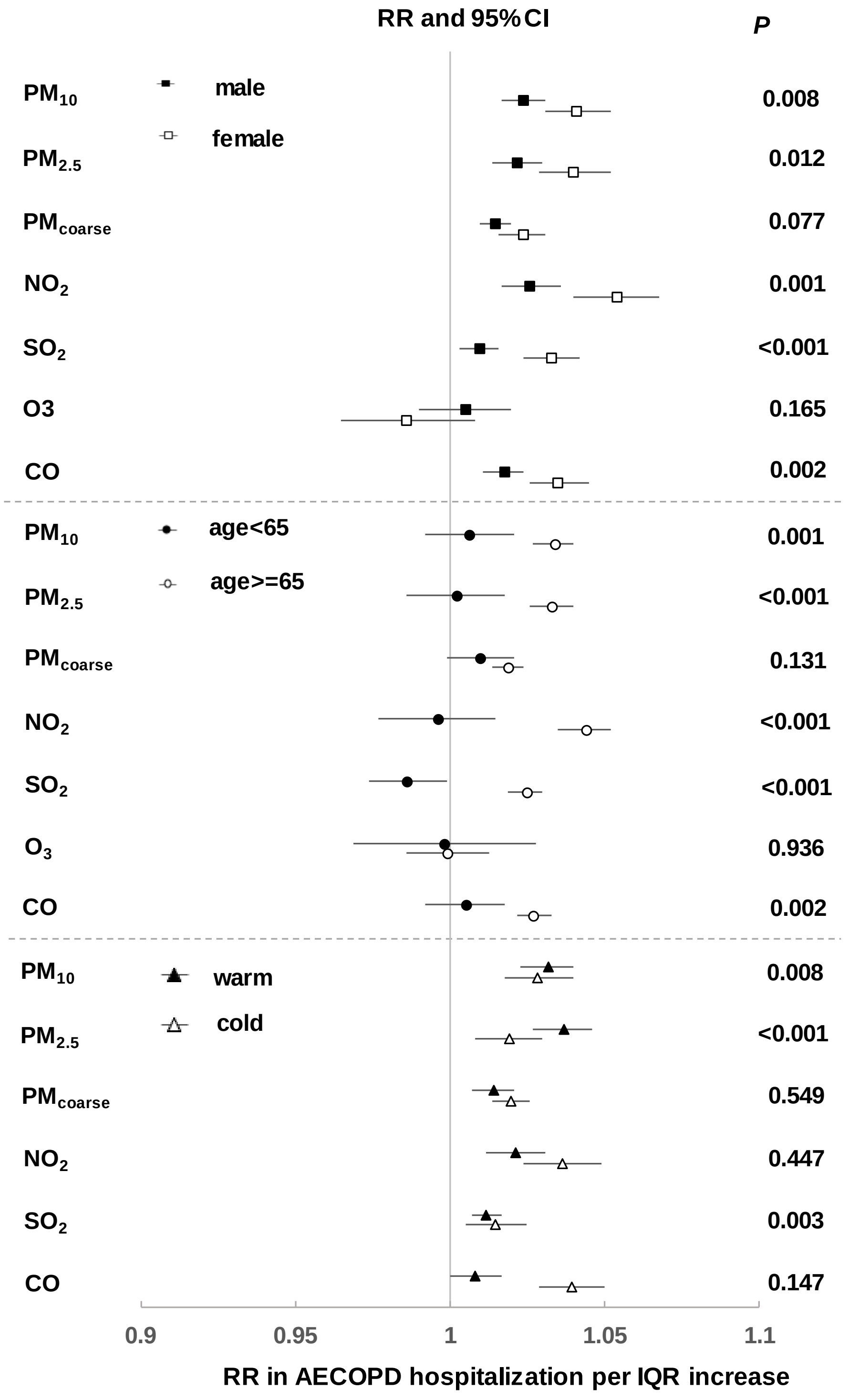

Figure S4 Associations at lag0 between air pollutants and AECOPD hospitalisation risk each year from 2013 to 2017

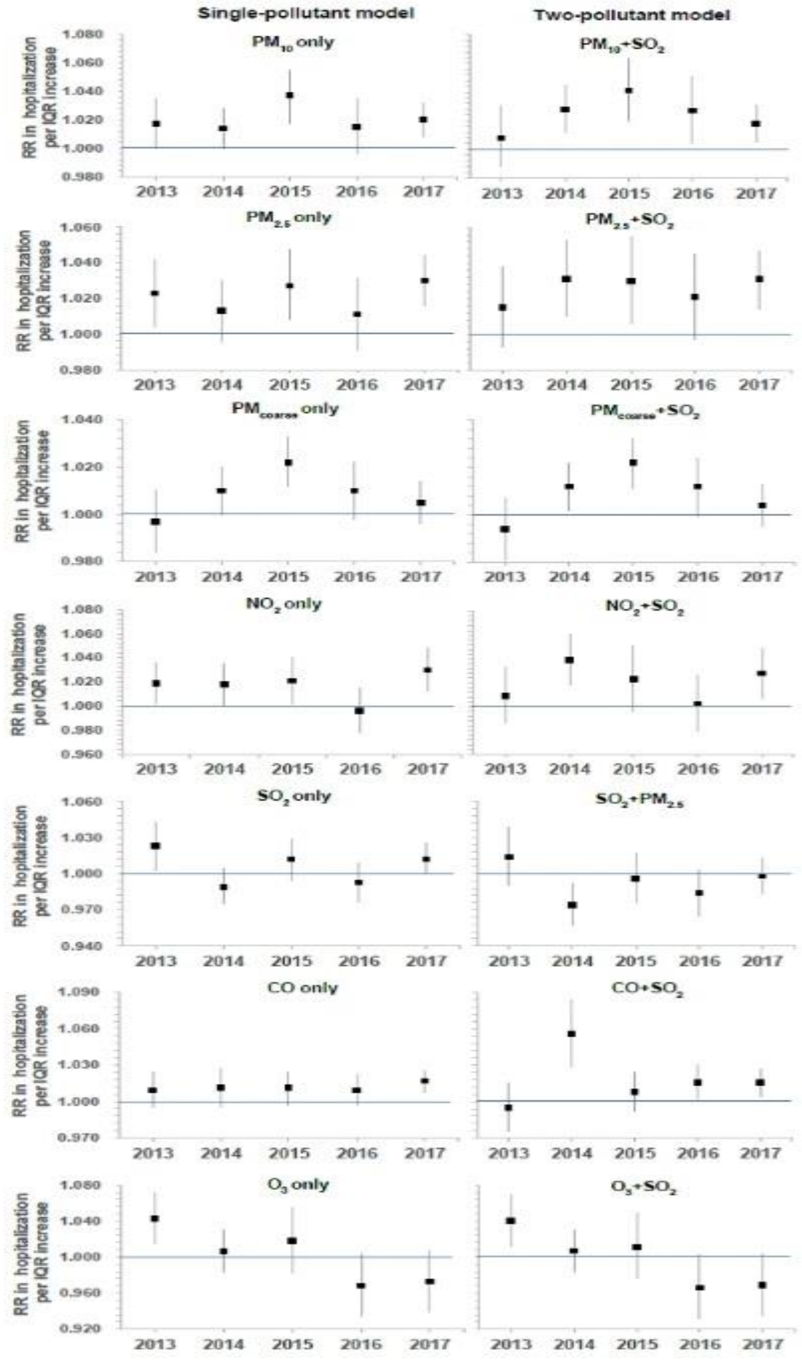

Supplemental Table 2. Matrix of spearman's correlation coefficients between daily air pollutants and meteorological factors in Beijing during 2013-2017.

	PM_{10}	$PM_{2.5}$	PM_{coarse}	NO_2	SO_2	O_3	CO	Average temperature	Relative humidity
PM_{10}	1								
$PM_{2.5}$	0.934**	1							
PM_{coarse}	0.576**	0.247**	1						
NO_2	0.729**	0.777**	0.200**	1					
SO_2	0.490**	0.546**	0.080**	0.638**	1				
O_3	-0.100**	-0.143**	0.056*	-0.386**	-0.289**	1			
CO	0.742**	0.826**	0.122**	0.812**	0.642**	-0.364**	1		
temperature	-0.118**	-0.160**	0.045	-0.352**	-0.464**	0.743**	-0.368**	1	
Relative humidity	0.307**	0.418**	-0.124**	0.237**	-0.088**	0.051*	0.345**	0.338**	1

Definition of abbreviations: PM_{10}, particulate matter<10 µm; $PM_{2.5}$, particulate matter<2.5 µm; PM_{coarse}, particulate matter 2.5–10 µm; NO_2, nitrogen dioxide; SO_2, sulfur dioxide; O_3, ozone; CO, carbon monoxide.

*. Correlation is significant at the 0.05 level (2-tailed).

**. Correlation is significant at the 0.01 level (2-tailed).

Supplemental Table 3.1 Relative risk and 95% CI per **interquartile-range** increment for each pollutant in AECOPD hospital admissions in single-pollutant and two-pollutant models at different lag days during 2013-2017.

		**Pollutant**	**lag**	**RR**	**lower95%CI**	**Upper 95%CI**
**single-pollutant model**	pm10	PM10	lag0	1.029	1.023	1.035
	pm10_lag1		lag1	1.023	1.018	1.029
	pm10_lag2		lag2	1.013	1.008	1.018
	pm10_lag3		lag3	1.008	1.003	1.013
	pm10_lag4		lag4	1.006	1.001	1.011
	pm10_mean3		lag0-2	1.028	1.022	1.034
	pm10_mean5		lag0-4	1.024	1.019	1.030
	pm25	PM2.5	lag0	1.028	1.021	1.034
	pm25_lag1		lag1	1.021	1.015	1.026
	pm25_lag2		lag2	1.012	1.007	1.018
	pm25_lag3		lag3	1.008	1.002	1.013
	pm25_lag4		lag4	1.004	0.999	1.010
	pm25_mean3		lag0-2	1.025	1.019	1.032
	pm25_mean5		lag0-4	1.021	1.016	1.027
	pmcoarse	PMcoarse	lag0	1.018	1.013	1.022
	pmcoarse_lag1		lag1	1.015	1.010	1.019
	pmcoarse_lag2		lag2	1.007	1.003	1.011
	pmcoarse_lag3		lag3	1.004	1.000	1.008
	pmcoarse_lag4		lag4	1.005	1.001	1.010
	pmcoarse_mean3		lag0-2	1.021	1.015	1.026
	pmcoarse_mean5		lag0-4	1.019	1.014	1.025
	no2	NO2	lag0	1.036	1.028	1.044
	no2_lag1		lag1	1.025	1.018	1.032
	no2_lag2		lag2	1.010	1.003	1.016
	no2_lag3		lag3	1.004	0.997	1.011

	no2_lag4		lag4	1.001	0.995	1.008
	no2_mean3		lag0-2	1.029	1.022	1.036
	no2_mean5		lag0-4	1.023	1.015	1.031
	so2	SO2	lag0	1.019	1.013	1.024
	so2_lag1		lag1	1.016	1.011	1.020
	so2_lag2		lag2	1.007	1.002	1.012
	so2_lag3		lag3	1.005	1.000	1.010
	so2_lag4		lag4	1.006	1.001	1.011
	so2_mean3		lag0-2	1.020	1.014	1.027
	so2_mean5		lag0-4	1.021	1.014	1.028
	co	CO	lag0	1.024	1.018	1.029
	co_lag1		lag1	1.017	1.013	1.021
	co_lag2		lag2	1.009	1.005	1.014
	co_lag3		lag3	1.004	1.000	1.008
	co_lag4		lag4	1.003	0.999	1.008
	co_mean3		lag0-2	1.022	1.017	1.027
	co_mean5		lag0-4	1.019	1.014	1.025
		**Pollutant**	**lag**	**RR**	**lower95%CI**	**Upper 95%CI**
**two-pollutant model**	pm10+so2	PM10	lag0	1.025	1.018	1.032
	pm10_lag1+so2_lag1		lag1	1.019	1.012	1.025
	pm10_lag2+so2_lag2		lag2	1.012	1.006	1.019
	pm10_lag3+so2_lag3		lag3	1.007	1.001	1.014
	pm10_lag4+so2_lag4		lag4	1.004	0.998	1.010
	pm10_mean3+so2_mean3		lag0-2	1.025	1.018	1.032
	pm10_mean5+so2_mean5		lag0-4	1.021	1.015	1.028
	pm25+so2	PM2.5	lag0	1.022	1.014	1.030
	pm25_lag1+so2_lag1		lag1	1.014	1.007	1.021
	pm25_lag2+so2_lag2		lag2	1.011	1.005	1.018

	pm25_lag3+so2_lag3		lag3	1.007	1.000	1.013
	pm25_lag4+so2_lag4		lag4	1.001	0.995	1.007
	pm25_mean3+so2_mean3		lag0-2	1.021	1.013	1.028
	pm25_mean5+so2_mean5		lag0-4	1.017	1.010	1.024
	pmcoarse+so2	PMcoarse	lag0	1.015	1.011	1.020
	pmcoarse_lag1+so2_lag1		lag1	1.013	1.008	1.017
	pmcoarse_lag2+so2_lag2		lag2	1.006	1.002	1.011
	pmcoarse_lag3+so2_lag3		lag3	1.003	0.999	1.008
	pmcoarse_lag4+so2_lag4		lag4	1.005	1.001	1.009
	pmcoarse_mean3+so2_mean3		lag0-2	1.018	1.013	1.023
	pmcoarse_mean5+so2_mean5		lag0-4	1.017	1.012	1.023
	no2+so2	NO2	lag0	1.029	1.019	1.039
	no2_lag1+so2_lag1		lag1	1.016	1.007	1.025
	no2_lag2+so2_lag2		lag2	1.006	0.997	1.014
	no2_lag3+so2_lag3		lag3	1.000	0.992	1.008
	no2_lag4+so2_lag4		lag4	0.994	0.986	1.002
	no2_mean3+so2_mean3		lag0-2	1.022	1.013	1.031
	no2_mean5+so2_mean5		lag0-4	1.015	1.006	1.024
	so2+pm25	SO2	lag0	1.009	1.002	1.015
	so2_lag1+pm25_lag1		lag1	1.009	1.003	1.015
	so2_lag2+pm25_lag2		lag2	1.001	0.995	1.007
	so2_lag3+pm25_lag3		lag3	1.001	0.996	1.007
	so2_lag4+pm25_lag4		lag4	1.005	1.000	1.011
	so2_mean3+pm25_mean3		lag0-2	1.009	1.001	1.016
	so2_mean5+pm25_mean5		lag0-4	1.010	1.002	1.019
	co+so2	CO	lag0	1.020	1.013	1.026
	co_lag1+so2_lag1		lag1	1.013	1.007	1.018
	co_lag2+so2_lag2		lag2	1.009	1.004	1.015

	co_lag3+so2_lag3		lag3	1.002	0.997	1.008
	co_lag4+so2_lag4		lag4	1.000	0.994	1.005
	co_mean3+so2_mean3		lag0-2	1.018	1.012	1.025
	co_mean5+so2_mean5		lag0-4	1.015	1.008	1.022

Supplemental Table 3.2 Relative risk and 95% CI per **10μg/m^3** increment for each pollutant in AECOPD hospital admissions in single-pollutant models at lag0 during 2013-2017.

single-pollutant model	Pollutant	unit	RR	Lower 95%CI	Upper 95%CI
	PM10	10	1.003	1.003	1.004
	PM2.5	10	1.004	1.003	1.005
	PMcoarse	10	1.007	1.005	1.009
	NO2	10	1.012	1.009	1.014
	SO2	10	1.012	1.009	1.016
	CO	1	1.030	1.023	1.036
	O3_warm season	10	1.003	1.001	1.004
	O3_cold season	10	0.990	0.986	0.994

Supplemental Table 4- Associations between per IQR higher air pollution exposure and daily AECOPD hospitalizations during 2013-2017 in Beijing

single-pollutant model					two-pollutant model				
**Pollutant**	**Lag**	**RR**	**95%CI**	**p**	**Pollutants**	**Lag**	**RR**	**95%CI**	**p**
**PM_{10}**	lag0	1.029	(1.023,1.035)	<0.001	**PM_{10}+ PMcoarse**	lag0	1.025	(1.016,1.033)	<0.001
	Lag1	1.023	(1.018,1.029)	<0.001		Lag1	1.019	(1.012,1.027)	<0.001
	lag2	1.013	(1.008,1.018)	<0.001		lag2	1.013	(1.006,1.019)	<0.001
	lag3	1.008	(1.003,1.013)	0.003		lag3	1.008	(1.001,1.015)	0.016
	lag4	1.006	(1.001,1.011)	0.026		lag4	1.003	(0.997,1.010)	0.334
	lag02	1.028	(1.022,1.034)	<0.001		lag02	1.023	(1.015,1.030)	<0.001
	lag04	1.024	(1.019,1.030)	<0.001		lag04	1.019	(1.012,1.027)	<0.001
**$PM_{2.5}$**	lag0	1.028	(1.021,1.034)	<0.001	**$PM_{2.5}$+ PMcoarse**	lag0	1.021	(1.013,1.028)	<0.001
	Lag1	1.021	(1.015,1.026)	<0.001		Lag1	1.016	(1.010,1.022)	<0.001
	lag2	1.012	(1.007,1.018)	<0.001		lag2	1.011	(1.005,1.016)	<0.001
	lag3	1.008	(1.002,1.013)	0.005		lag3	1.007	(1.001,1.012)	0.016
	lag4	1.004	(0.999,1.010)	0.121		lag4	1.003	(0.997,1.008)	0.334
	lag02	1.025	(1.019,1.032)	<0.001		lag02	1.019	(1.012,1.025)	<0.001
	lag04	1.021	(1.016,1.027)	<0.001		lag04	1.016	(1.010,1.022)	<0.001
PM_{coarse}	lag0	1.018	(1.013,1.022)	<0.001	**PMcoarse + PM_{10}**	lag0	1.005	(0.999,1.011)	0.136
	Lag1	1.015	(1.010,1.019)	<0.001		Lag1	1.005	(0.999,1.011)	0.086
	lag2	1.007	(1.003,1.011)	0.002		lag2	1.001	(0.995,1.006)	0.822
	lag3	1.004	(1.000,1.008)	0.075		lag3	1.000	(0.995,1.005)	0.980
	lag4	1.005	(1.001,1.010)	0.013		lag4	1.004	(0.999,1.009)	0.145
	lag02	1.021	(1.015,1.026)	<0.001		lag02	1.008	(1.001,1.014)	0.028
	lag04	1.019	(1.014,1.025)	<0.001		lag04	1.008	(1.001,1.015)	0.033
					**PMcoarse + PM2.5**	lag0	1.012	(1.007,1.017)	<0.001

		Lag1	1.011	(1.006,1.015)	<0.001
		lag2	1.004	(1.000,1.009)	0.061
		lag3	1.002	(0.998,1.007)	0.294
		lag4	1.005	(1.000,1.009)	0.031
		lag02	1.014	(1.009,1.020)	<0.001
		lag04	1.014	(1.008,1.020)	<0.001
	**PMcoarse + NO2**	lag0	1.013	(1.008,1.017)	<0.001
		Lag1	1.011	(1.006,1.016)	<0.001
		lag2	1.006	(1.001,1.010)	0.011
		lag3	1.004	(0.999,1.008)	0.118
		lag4	1.005	(1.001,1.010)	0.014
		lag02	1.016	(1.010,1.021)	<0.001
		lag04	1.016	(1.010,1.021)	<0.001
	**PMcoarse + O3**	lag0	1.017	(1.013,1.022)	<0.001
		Lag1	1.015	(1.010,1.019)	<0.001
		lag2	1.008	(1.003,1.012)	0.001
		lag3	1.005	(1.000,1.009)	0.039
		lag4	1.006	(1.002,1.010)	0.007
		lag02	1.021	(1.016,1.026)	<0.001
		lag04	1.020	(1.014,1.025)	<0.001
	**PMcoarse + CO**	lag0	1.013	(1.008,1.017)	<0.001
		Lag1	1.011	(1.006,1.016)	<0.001
		lag2	1.005	(1.001,1.010)	0.022
		lag3	1.003	(0.999,1.008)	0.143
		lag4	1.005	(1.001,1.009)	0.023
		lag02	1.015	(1.010,1.021)	<0.001

						lag04	1.015	(1.010,1.021)	<0.001
**NO_2**	lag0	1.036	(1.028,1.044)	<0.001	**NO_2+ PMcoarse**	lag0	1.028	(1.020,1.037)	<0.001
	Lag1	1.025	(1.018,1.032)	<0.001		Lag1	1.020	(1.012,1.027)	<0.001
	lag2	1.010	(1.003,1.016)	0.004		lag2	1.008	(1.001,1.014)	0.029
	lag3	1.004	(0.997,1.011)	0.239		lag3	1.003	(0.996,1.010)	0.414
	lag4	1.001	(0.995,1.008)	0.742		lag4	0.999	(0.993,1.006)	0.868
	lag02	1.029	(1.022,1.036)	<0.001		lag02	1.021	(1.014,1.029)	<0.001
	lag04	1.023	(1.015,1.031)	<0.001		lag04	1.016	(1.007,1.024)	<0.001
**CO**	lag0	1.024	(1.018,1.029)	<0.001	**CO+ PMcoarse**	lag0	1.019	(1.013,1.024)	<0.001
	Lag1	1.017	(1.013,1.021)	<0.001		Lag1	1.014	(1.010,1.019)	<0.001
	lag2	1.009	(1.005,1.014)	<0.001		lag2	1.008	(1.004,1.013)	<0.001
	lag3	1.004	(1.000,1.008)	0.068		lag3	1.003	(0.999,1.008)	0.127
	lag4	1.003	(0.999,1.008)	0.162		lag4	1.002	(0.998,1.007)	0.314
	lag02	1.022	(1.017,1.027)	<0.001		lag02	1.017	(1.012,1.023)	<0.001
	lag04	1.019	(1.014,1.025)	<0.001		lag04	1.015	(1.009,1.021)	<0.001
**SO_2**	lag0	1.019	(1.013,1.024)	<0.001	**SO_2+ PM_{10}**	lag0	1.007	(1.001,1.013)	0.015
	Lag1	1.016	(1.011,1.020)	<0.001		Lag1	1.007	(1.002,1.013)	0.010
	lag2	1.007	(1.002,1.012)	0.005		lag2	1.001	(0.996,1.007)	0.682
	lag3	1.005	(1.000,1.010)	0.056		lag3	1.001	(0.996,1.007)	0.630
	lag4	1.006	(1.001,1.011)	0.019		lag4	1.004	(0.999,1.010)	0.151
	lag02	1.020	(1.014,1.027)	<0.001		lag02	1.007	(1.000,1.014)	0.056
	lag04	1.021	(1.014,1.028)	<0.001		lag04	1.008	(1.000,1.017)	0.046
					**SO_2+ PMcoarse**	lag0	1.015	(1.010,1.021)	<0.001
						Lag1	1.014	(1.009,1.018)	<0.001
						lag2	1.006	(1.001,1.011)	0.014

		lag3	1.004	(0.999,1.009)	0.086
		lag4	1.005	(1.000,1.010)	0.034
		lag02	1.016	(1.010,1.023)	<0.001
		lag04	1.017	(1.010,1.025)	<0.001
	**SO_2+ NO_2**	lag0	1.007	(1.001,1.013)	0.034
		Lag1	1.009	(1.002,1.015)	0.006
		lag2	1.004	(0.998,1.010)	0.188
		lag3	1.005	(0.999,1.011)	0.132
		lag4	1.009	(1.002,1.015)	0.006
		lag02	1.009	(1.002,1.017)	0.015
		lag04	1.013	(1.005,1.022)	0.002
	**SO_2+ CO**	lag0	1.006	(1.000,1.013)	0.067
		Lag1	1.007	(1.001,1.013)	0.022
		lag2	1.001	(0.995,1.007)	0.833
		lag3	1.003	(0.997,1.009)	0.311
		lag4	1.006	(1.000,1.012)	0.059
		lag02	1.007	(1.000,1.015)	0.051
		lag04	1.011	(1.002,1.019)	0.014

Supplemental Table 5.1 – Sensitivity analysis (a) : Relative risk and 95% CI per **interquartile-range** increment for each pollutant in AECOPD hospital admissions in single-pollutant models at different lag days during 2013-2017: **calendar time was removed from the model**

**Pollutant**	**lag0**		**lag1**		**lag2**		**lag3**		**lag4**		**lag0-2**		**lag0-4**	
	**RR（95% CI）**	**p**	**RR（95% CI）**	**p**	**RR（95% CI）**	**p**	**RR（95% CI）**	**p**	**RR（95% CI）**	**p**	**RR（95% CI）**	**p**	**RR（95% CI）**	**p**
**PM_{10} (μ g/m³)**	1.067 (1.061,1.073)	<0.001	1.053 (1.047,1.059)	<0.001	1.036 (1.031,1.041)	<0.001	1.031 (1.026,1.036)	<0.001	1.03 (1.024,1.035)	<0.001	1.067 (1.061,1.072)	<0.001	1.064 (1.059,1.07)	<0.001
**$PM_{2.5}$(μ g/m³)**	1.078 (1.072,1.085)	<0.001	1.055 (1.049,1.06)	<0.001	1.039 (1.034,1.045)	<0.001	1.034 (1.029,1.039)	<0.001	1.032 (1.027,1.037)	<0.001	1.071 (1.065,1.077)	<0.001	1.069 (1.063,1.074)	<0.001
**PM_{coarse} (μ g/m³)**	1.021 (1.017,1.026)	<0.001	1.019 (1.015,1.023)	<0.001	1.009 (1.005,1.013)	<0.001	1.005 (1.001,1.009)	0.026	1.006 (1.002,1.01)	0.007	1.025 (1.02,1.03)	<0.001	1.022 (1.017,1.027)	<0.001
**NO_2(μ g/m³)**	1.105 (1.097,1.113)	<0.001	1.076 (1.069,1.083)	<0.001	1.053 (1.046,1.059)	<0.001	1.048 (1.041,1.054)	<0.001	1.044 (1.038,1.051)	<0.001	1.098 (1.091,1.105)	<0.001	1.102 (1.094,1.109)	<0.001
**SO_2(μ g/m³)**	1.080 (1.075,1.084)	<0.001	1.068 (1.064,1.072)	<0.001	1.062 (1.058,1.066)	<0.001	1.062 (1.058,1.066)	<0.001	1.062 (1.057,1.066)	<0.001	1.094 (1.089,1.099)	<0.001	1.111 (1.105,1.117)	<0.001
**CO(mg/m³)**	1.059 (1.054,1.064)	<0.001	1.041 (1.036,1.045)	<0.001	1.029 (1.024,1.033)	<0.001	1.023 (1.019,1.027)	<0.001	1.024 (1.02,1.028)	<0.001	1.055 (1.051,1.06)	<0.001	1.058 (1.052,1.063)	<0.001
**O3_warm**	0.973 (0.958,0.988)	0.001	0.978 (0.964,0.992)	0.002	0.971 (0.958,0.984)	<0.001	0.968 (0.955,0.981)	<0.001	0.968 (0.956,0.981)	<0.001	0.968 (0.954,0.981)	<0.001	0.958 (0.944,0.971)	<0.001
**O3_cold**	0.961 (0.947,0.976)	<0.001	0.979 (0.966,0.993)	0.003	1.001 (0.988,1.015)	0.829	0.996 (0.984,1.008)	0.506	0.995 (0.983,1.007)	0.421	0.974 (0.958,0.99)	0.002	0.978 (0.961,0.995)	0.013

Supplemental Table 5.2 – Sensitivity analysis (b) : Relative risk and 95% CI per **interquartile-range** increment for each pollutant in AECOPD hospital admissions in single-pollutant models at different lag days during 2013-2017: **calendar time was removed from the model whilst an interaction term 'exposure by season' was added**

Pollutant	lag0		lag1		lag2		lag3		lag4		lag0-2		lag0-4	
	RR（95% CI）	p	RR（95% CI）	p	RR（95% CI）	p	RR（95% CI）	p	RR（95% CI）	p	RR（95% CI）	p	RR（95% CI）	p
PM_{10} (μg/m^3)	1.083 (1.073,1.093)	<0.001	1.072 (1.061,1.083)	<0.001	1.051 (1.042,1.061)	<0.001	1.049 (1.039,1.059)	<0.001	1.044 (1.034,1.054)	<0.001	1.099 (1.088,1.11)	<0.001	1.103 (1.091,1.114)	<0.001
$PM_{2.5}$(μg/m^3)	1.103 (1.092,1.115)	<0.001	1.074 (1.063,1.085)	<0.001	1.057 (1.047,1.068)	<0.001	1.054 (1.044,1.064)	<0.001	1.045 (1.035,1.055)	<0.001	1.101 (1.09,1.113)	<0.001	1.103 (1.092,1.114)	<0.001
PM_{coarse} (μg/m^3)	1.025 (1.018,1.032)	<0.001	1.025 (1.017,1.032)	<0.001	1.014 (1.008,1.021)	<0.001	1.012 (1.006,1.019)	<0.001	1.015 (1.008,1.021)	<0.001	1.035 (1.026,1.043)	<0.001	1.035 (1.027,1.044)	<0.001
NO_2(μg/m^3)	1.13 (1.115,1.146)	<0.001	1.103 (1.088,1.118)	<0.001	1.09 (1.075,1.105)	<0.001	1.091 (1.076,1.106)	<0.001	1.086 (1.071,1.101)	<0.001	1.137 (1.122,1.153)	<0.001	1.152 (1.136,1.168)	<0.001
SO_2(μg/m^3)	1.111 (1.097,1.125)	<0.001	1.098 (1.084,1.112)	<0.001	1.089 (1.075,1.104)	<0.001	1.081 (1.067,1.096)	<0.001	1.091 (1.078,1.105)	<0.001	1.155 (1.137,1.173)	<0.001	1.185 (1.164,1.207)	<0.001
CO(mg/m^3)	1.072 (1.061,1.083)	<0.001	1.055 (1.044,1.067)	<0.001	1.043 (1.032,1.054)	<0.001	1.028 (1.017,1.039)	<0.001	1.03 (1.019,1.041)	<0.001	1.082 (1.07,1.095)	<0.001	1.088 (1.073,1.102)	<0.001

P-value for the interaction term

Pollutant	lag0	lag1	lag2	lag3	lag4	lag0-2	lag0-4
season*PM_{10} (μg/m^3)	<0.001	<0.001	<0.001	<0.001	<0.001	<0.001	<0.001
season*$PM_{2.5}$(μg/m^3)	<0.001	<0.001	<0.001	<0.001	0.003	<0.001	<0.001
season*PM_{coarse} (μg/m^3)	0.043	0.020	0.013	0.001	<0.001	<0.001	<0.001
season*NO_2(μg/m^3)	<0.001	<0.001	<0.001	<0.001	<0.001	<0.001	<0.001
season*SO_2(μg/m^3)	<0.001	<0.001	<0.001	0.003	<0.001	<0.001	<0.001
season*CO(mg/m^3)	0.007	0.004	0.005	0.289	0.233	<0.001	<0.001

Supplemental Table 5.3 – Sensitivity analysis (c) : Relative risk and 95% CI per **interquartile-range** increment for each pollutant in AECOPD hospital admissions in single-pollutant models at different lag days during 2013-2017: **to increase degree of freedom from three to six for temperature and relative humidity**

Pollutant	**lag0**		**lag1**		**lag2**		**lag3**		**lag4**		**lag0-2**		**lag0-4**	
	**RR（95% CI）**	**p**	**RR（95% CI）**	**p**	**RR（95% CI）**	**p**	**RR（95% CI）**	**p**	**RR（95% CI）**	**p**	**RR（95% CI）**	**p**	**RR（95% CI）**	**p**
**PM_{10} (μg/m^3)**	1.029 (1.023，1.035）	<0.001	1.022 (1.016，1.028）	<0.001	1.012 (1.007，1.017）	<0.001	1.007 (1.002，1.012）	0.010	1.005 (1，1.01）	0.064	1.027 (1.021，1.033）	<0.001	1.023 (1.017，1.029）	<0.001
**$PM_{2.5}$(μg/m^3)**	1.027 (1.02，1.033）	<0.001	1.019 (1.013，1.025）	<0.001	1.011 (1.006，1.017）	<0.001	1.007 (1.001，1.012）	0.016	1.003 (0.998，1.008）	0.287	1.024 (1.018，1.03）	<0.001	1.02 (1.014，1.026）	<0.001
**PM_{coarse} (μg/m^3)**	1.018 (1.014，1.023）	<0.001	1.015 (1.01，1.019）	<0.001	1.006 (1.002，1.011）	0.005	1.004 (0.999，1.008）	0.110	1.006 (1.001，1.01）	0.009	1.021 (1.016，1.026）	<0.001	1.019 (1.014，1.025）	<0.001
**NO_2(μg/m^3)**	1.035 (1.027，1.043）	<0.001	1.022 (1.015，1.029）	<0.001	1.008 (1.002，1.015）	0.016	1.003 (0.996，1.01）	0.378	1.001 (0.994，1.007）	0.842	1.027 (1.019，1.035）	<0.001	1.021 (1.013，1.029）	<0.001
**SO_2(μg/m^3)**	1.017 (1.012，1.023）	<0.001	1.014 (1.009，1.019）	<0.001	1.005 (1，1.01）	0.039	1.004 (0.999，1.009）	0.128	1.005 (1，1.01）	0.059	1.018 (1.012，1.025）	<0.001	1.019 (1.011，1.026）	<0.001
**CO(mg/m^3)**	1.024 (1.018，1.029）	<0.001	1.016 (1.011，1.02）	<0.001	1.008 (1.004，1.013）	<0.001	1.003 (0.998，1.007）	0.221	1.002 (0.997，1.006）	0.416	1.021 (1.016，1.026）	<0.001	1.018 (1.012，1.024）	<0.001
**O3_warm**	1.024 (1.007，1.041）	0.005	1.018 (1.003，1.034）	0.018	1.006 (0.992，1.02）	0.423	1.002 (0.988，1.016）	0.780	0.998 (0.984，1.012）	0.773	1.02 (1.005，1.036）	0.011	1.014 (0.998，1.03）	0.081

**O3_cold**	0.952 (0.936，0.969）	<0.001	0.971 (0.956，0.986）	<0.001	0.99 (0.977，1.004）	0.180	0.987 (0.974，1）	0.059	0.99 (0.977，1.003）	0.122	0.96 (0.941，0.978）	<0.001	0.962 (0.943，0.982）	<0.001

Supplemental Table 5.4 – Sensitivity analysis (d) : Relative risk and 95% CI per **interquartile-range** increment for each pollutant in AECOPD hospital admissions in single-pollutant models at different lag days during 2013-2017: **to model moving averages lag0-4 of temperature and humidity**

**Pollutant**	**lag0**		**lag1**		**lag2**		**lag3**		**lag4**		**lag0-2**		**lag0-4**	
	**RR（95% CI）**	**p**	**RR（95% CI）**	**p**	**RR（95% CI）**	**p**	**RR（95% CI）**	**p**	**RR（95% CI）**	**p**	**RR（95% CI）**	**p**	**RR（95% CI）**	**p**
**PM_{10} (μ 0/m^3)**	1.015 (1.009，1.02)	<0.001	1.015 (1.009，1.02）	<0.001	1.014 (1.008，1.019）	<0.001	1.012 (1.007，1.018）	<0.001	1.011 (1.005，1.016）	<0.001	1.02 (1.014，1.026）	<0.001	1.023 (1.017，1.03）	<0.001
**$PM_{2.5}$(μ ./m^3)**	1.009 (1.004，1.015)	0.001	1.01 (1.004，1.016）	0.001	1.012 (1.006，1.018）	<0.001	1.011 (1.006，1.017）	<0.001	1.009 (1.003，1.014）	0.002	1.015 (1.009，1.021）	<0.001	1.02 (1.014，1.027）	<0.001
**PM_{coarse} (μ o/m^3)**	1.016 (1.011，1.02)	<0.001	1.014 (1.01，1.019）	<0.001	1.009 (1.005，1.014）	<0.001	1.007 (1.003，1.011）	0.002	1.008 (1.004，1.012）	<0.001	1.021 (1.016，1.026）	<0.001	1.022 (1.017，1.028）	<0.001
**NO_2(μ O/m^3)**	1.016 (1.009，1.022)	<0.001	1.009 (1.002，1.016）	0.008	1.005 (0.998，1.012）	0.127	1.006 (0.999，1.013）	0.095	1.006 (0.999，1.013）	0.071	1.014 (1.007，1.021）	<0.001	1.017 (1.009，1.026）	<0.001
**SO_2(μ O/m^3)**	1.011 (1.006，1.016)	<0.001	1.008 (1.003，1.013）	0.001	1.002 (0.997，1.007）	0.356	1.003 (0.998，1.008）	0.258	1.006 (1.001，1.011）	0.014	1.011 (1.005，1.018）	<0.001	1.014 (1.006，1.021）	<0.001
**CO(mg/m^3)**	1.009 (1.004，1.013)	<0.001	1.008 (1.004，1.013）	<0.001	1.008 (1.003，1.012）	0.002	1.005 (1，1.009）	0.049	1.005 (1.001，1.01）	0.028	1.012 (1.007，1.018）	<0.001	1.016 (1.009，1.022）	<0.001

**O3_warm**	1.03 (1.015，1.045)	<0.001	1.029 (1.013，1.044）	<0.001	1.016 (1.001，1.031）	0.042	1.016 (1.001，1.031）	0.040	1.012 (0.997，1.027）	0.109	1.035 (1.019，1.051）	<0.001	1.04 (1.022，1.058）	<0.001
**O3_cold**	0.969 (0.954，0.984)	<0.001	0.982 (0.966，0.998）	0.027	1.000 (0.984，1.017）	0.974	0.993 (0.978，1.009）	0.406	0.992 (0.977，1.007）	0.282	0.966 (0.945，0.987）	0.002	0.956 (0.929，0.983）	0.002

Supplemental Table 5.5 - Sensitivity analysis for 2013-2017 based on the 1702-days dataset with complete data on all pollutants.

Single-pollutant model										
	lag0		lag2		lag4		lag0-2		lag0-4	
	RR	95% CI	RR	95% CI	RR	95% CI	RR	95% CI	RR	95% CI
**PM_{10} (μg/m^3)**	1.034	(1.027,1.040)	1.014	(1.009,1.020)	1.006	(1.000,1.011)	1.042	(1.034,1.050)	1.041	(1.032,1.051)
**p**		<0.001		<0.001		0.049		<0.001		<0.001
**$PM_{2.5}$(μg/m^3)**	1.029	(1.022,1.036)	1.012	(1.007,1.018)	1.003	(0.998,1.008)	1.035	(1.027,1.043)	1.033	(1.024,1.043)
**p**		<0.001		<0.001		0.277		<0.001		<0.001
**PM_{coarse} (μg/m^3)**	1.021	(1.016,1.025)	1.007	(1.003,1.012)	1.006	(1.002,1.010)	1.027	(1.021,1.033)	1.027	(1.020,1.035)
**p**		<0.001		0.001		0.006		<0.001		<0.001
**NO_2(μg/m^3)**	1.034	(1.026,1.043)	1.008	(1.001,1.015)	0.999	(0.992,1.006)	1.037	(1.027,1.047)	1.029	(1.017,1.040)
**p**		<0.001		0.017		0.733		<0.001		<0.001
**SO_2(μg/m^3)**	1.018	(1.013,1.024)	1.007	(1.002,1.012)	1.001	(0.996,1.006)	1.021	(1.014,1.027)	1.018	(1.011,1.026)
**p**		<0.001		0.005		0.610		<0.001		<0.001
**CO(mg/m^3)**	1.025	(1.019,1.030)	1.009	(1.005,1.014)	1.002	(0.997,1.006)	1.027	(1.021,1.033)	1.024	(1.017,1.032)
**p**		<0.001		<0.001		0.437		<0.001		<0.001

Two-pollutant model*										
	lag0		lag2		lag4		lag0-2		lag0-4	
	RR	95% CI	RR	95% CI	RR	95% CI	RR	95% CI	RR	95% CI
**PM_{10} ($\mu g/m^3$)**	1.03	(1.023,1.038)	1.014	(1.007,1.020)	1.007	(1.000,1.013)	1.039	(1.029,1.048)	1.039	(1.028,1.050)
**p**		<0.001		<0.001		0.047		<0.001		<0.001
**$PM_{2.5}$($\mu g/m^3$)**	1.024	(1.015,1.032)	1.011	(1.005,1.018)	1.003	(0.997,1.010)	1.029	(1.020,1.039)	1.029	(1.018,1.040)
**p**		<0.001		0.001		0.334		<0.001		<0.001
**PM_{coarse} ($\mu g/m^3$)**	1.019	(1.014,1.023)	1.007	(1.002,1.011)	1.006	(1.002,1.010)	1.025	(1.018,1.031)	1.025	(1.018,1.033)
**p**		<0.001		0.002		0.006		<0.001		<0.001
**NO_2($\mu g/m^3$)**	1.027	(1.017,1.038)	1.003	(0.995,1.012)	0.996	(0.988,1.005)	1.027	(1.015,1.039)	1.02	(1.006,1.033)
**p**		<0.001		0.438		0.411		<0.001		0.004
**SO_2($\mu g/m^3$)**	1.007	(1.001,1.014)	1.001	(0.995,1.007)	1.000	(0.994,1.006)	1.008	(1.000,1.015)	1.006	(0.998,1.015)
**p**		0.027		0.662		0.910		0.044		0.143
**CO(mg/m^3)**	1.022	(1.015,1.029)	1.009	(1.003,1.015)	1.002	(0.996,1.007)	1.023	(1.015,1.031)	1.021	(1.012,1.029)
**p**		<0.001		0.002		0.556		<0.001		<0.001

* Further adjusted for SO_2 in the models of PM_{10}, $PM_{2.5}$, PMcoarse, NO_2, O_3 and CO; and further adjusted for $PM_{2.5}$ in the model of SO_2.

Supplemental Table 6- Comparisons of our study findings with those presented in a previous meta-analysis by Moore et el. published in 2016

	Moore et al 2016					Liang et al 2018
	**Global**	**North America**	**Europe**	**Asia**	**Study locations and periods in Asia**	**Beijing (2013-2017), Lag0**
**PM_{10} (per 10μg/m^3)**	1.01 [1.00-1.01]	1.00[1.00-1.01]	1.01[1.00-1.01]	1.02[1.01-1.03] (N=5)	Hong Kong (1998-2007)/Lanzhou, China (2001-2005)/Nepal(2003-2004)/New Zealand(1988-1998)	1.003 [1.003-1.004]
**$PM_{2.5}$ (per 10μg/m^3)**	1.03 [1.01-1.05]	1.01[0.99-1.03]	1.02[0.99-1.04]	1.04[1.00-1.08] (N=3)	Hong Kong (2000-2007)/Taiwan (2006-2010)	1.004 [1.003-1.005]
**NO_2 (per 10μg/m^3)**	1.03 [1.02-1.05]	1.00 [1.00-1.01]	1.01 [1.00-1.02]	1.07 [1.01-1.13] (N=6)	Hong Kong (1998-2007)/Taiwan(1996-2003)/Lanzhou, China(2001-2005)	1.012 [1.009-1.014]
**SO_2 (per 10μg/m^3)**	1.00[1.00-1.01]	1.00[0.99-1.01]	1.00[1.00-1.01]	1.03[1.00-1.06] (N=5)	Hong Kong (1998-2007)/Taiwan(1996-2003)/Lanzhou, China(2001-2005)	1.012[1.009-1.016]
**CO (per 1mg/m^3)**	1.02 [1.01-1.03]	1.02[1.01-1.03]	1.04[1.02-1.06]	N/A	N/A	1.030 [1.023-1.036]
**O_3 (per 10μg/m^3)**	1.02 [1.01-1.03]	1.01[1.00-1.01]	1.01 [0.99-1.04]	1.04 [1.03-1.05] (N=3)	Hong Kong (1998-2007)/Taiwan (1996-2003)	1.003 [1.001-1.005] (warm season)
						0.994 [0.992-0.996] (cold season)

Reference: Moore E, Chatzidiakou L, Kuku MO, Jones RL, Smeeth L, Beevers S, Kelly FJ, Barratt B, Quint JK. Global Associations between Air Pollutants and Chronic Obstructive Pulmonary Disease Hospitalizations. A Systematic Review. Ann Am Thorac Soc 2016; 13: 1814-1827.

Supplemental Table 7- Comparisons of our study findings on $PM_{2.5}$ with previous studies conducted in Beijing

	Liang et al, 2018	Xu et al, 2016	Tian et al, 2018
**Study periods**	2013/01/18-2017/12/31	2013/01/01-2013/12/31	2010-2012
**Outcome**	AECOPD (ICD-10: J44.0-J44.9)	AECOPD (ICD-10: J44.103, J44.901)	COPD (ICD-10: J40–J44)
**Populations**	all Beijing residents	patients admitted to 10 general hospitals in urban areas in Beijing	all working or retired employees who are covered by basic medical insurance in Beijing
**$PM_{2.5}$ data**	averaged from 35 stations all over the city	17 ambient air quality monitoring stations located in urban areas in Beijing city	The monitoring station from US embassy
**Results as of per 10 μg/m³ increase of $PM_{2.5}$ on lag0**	0.4% (95%CI: 0.3%-0.5%) (Year 2013-2017)	1.46% (95%CI: 0.13%-2.79%)	0.26% (95%CI: 0.24%-0.28%) (Outpatients visits)<br>0.67% (95%CI: 0.56%-0.73%) (Inpatient visits)

References: Xu Q, Li X, Wang S, Wang C, Huang F, Gao Q, Wu L, Tao L, Guo J, Wang W, Guo X. Fine Particulate Air Pollution and Hospital Emergency Room Visits for Respiratory Disease in Urban Areas in Beijing, China, in 2013. *PLoS One* 2016; 11: e0153099.
Tian Y, Xiang X, Juan J, Song J, Cao Y, Huang C, Li M, Hu Y. Short-term effects of ambient fine particulate matter pollution on hospital visits for chronic obstructive pulmonary disease in Beijing, China. *Environ Health* 2018; 17: 21-018-0369-y.
